# DigiMOF: A Database of Metal–Organic Framework Synthesis Information Generated via Text Mining

**DOI:** 10.1021/acs.chemmater.3c00788

**Published:** 2023-05-18

**Authors:** Lawson T. Glasby, Kristian Gubsch, Rosalee Bence, Rama Oktavian, Kesler Isoko, Seyed Mohamad Moosavi, Joan L. Cordiner, Jason C. Cole, Peyman Z. Moghadam

**Affiliations:** † Department of Chemical and Biological Engineering, The University of Sheffield, Sheffield S1 3JD, U.K.; ‡ Department of Chemical Engineering, University College London, London WC1E 7JE, U.K.; § Chemical Engineering & Applied Chemistry, University of Toronto, Toronto, Ontario M5S 3E5, Canada; ∥ Cambridge Crystallographic Data Centre, Cambridge CB2 1EZ, U.K.

## Abstract

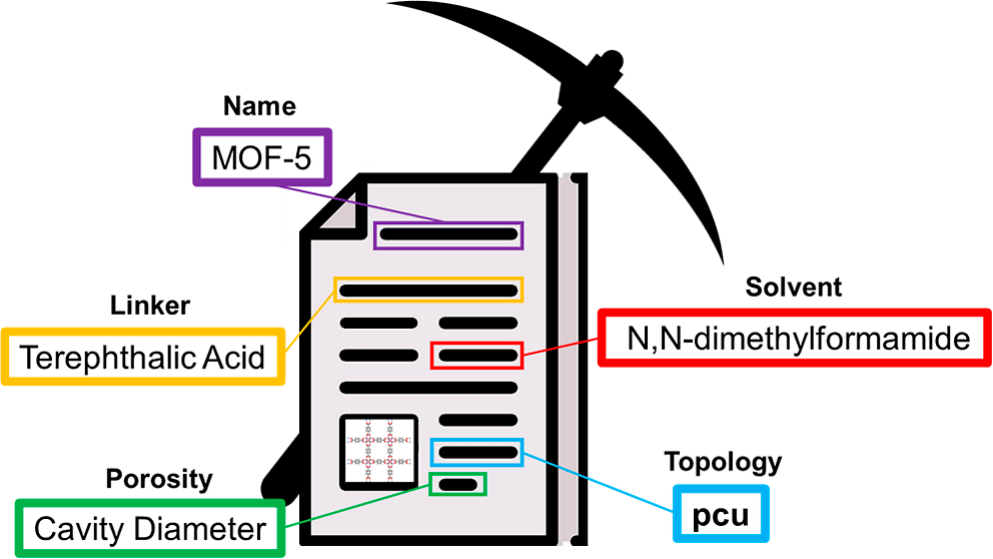

The vastness of materials space, particularly that which is concerned with metal–organic frameworks (MOFs), creates the critical problem of performing efficient identification of promising materials for specific applications. Although high-throughput computational approaches, including the use of machine learning, have been useful in rapid screening and rational design of MOFs, they tend to neglect descriptors related to their synthesis. One way to improve the efficiency of MOF discovery is to data-mine published MOF papers to extract the materials informatics knowledge contained within journal articles. Here, by adapting the chemistry-aware natural language processing tool, ChemDataExtractor (CDE), we generated an open-source database of MOFs focused on their synthetic properties: the DigiMOF database. Using the CDE web scraping package alongside the Cambridge Structural Database (CSD) MOF subset, we automatically downloaded 43,281 unique MOF journal articles, extracted 15,501 unique MOF materials, and text-mined over 52,680 associated properties including the synthesis method, solvent, organic linker, metal precursor, and topology. Additionally, we developed an alternative data extraction technique to obtain and transform the chemical names assigned to each CSD entry in order to determine linker types for each structure in the CSD MOF subset. This data enabled us to match MOFs to a list of known linkers provided by Tokyo Chemical Industry UK Ltd. (TCI) and analyze the cost of these important chemicals. This centralized, structured database reveals the MOF synthetic data embedded within thousands of MOF publications and contains further topology, metal type, accessible surface area, largest cavity diameter, pore limiting diameter, open metal sites, and density calculations for all 3D MOFs in the CSD MOF subset. The DigiMOF database and associated software are publicly available for other researchers to rapidly search for MOFs with specific properties, conduct further analysis of alternative MOF production pathways, and create additional parsers to search for additional desirable properties.

## 1 Introduction

Metal–organic frameworks (MOFs) are a class of crystalline materials consisting of a lattice of metal ions co-ordinately bonded by organic linkers. MOFs are well known for their high surface areas and exceptionally tunable properties, which enable their potential application in areas including gas storage,^1−6^ sensing,^7−10^ separations,^11−15^ drug delivery,^16−18^ and catalysis.^19−23^ Since the first MOFs were synthesized in the 1990s, thousands of MOFs have been produced at a laboratory scale. As of 2023, more than 100,000 MOF structures have been reported in the Cambridge Structural Database (CSD).^24,25^ The sheer volume of distinct real MOF materials poses significant challenges for screening and isolating the best candidates for a given application: a typical problem of finding a needle in a haystack. To some extent, this has been counteracted by the use of high-throughput computational screening and machine learning (ML) for the elucidation of structure–property relationships, in particular for gas adsorption and separation properties of MOFs.^26−32^ Given that these screening methods tend to neglect synthesis data, the identification of economical and sustainable synthesis routes has remained largely a manual process, and clearly, relying on experimental trial-and-error and serendipity to develop MOFs is costly, slow, and unreliable. While ML has so far been successfully applied to MOF synthesis using failed experimental data,^33^ to address these challenges, we propose the use of high-throughput text mining to collect MOF synthesis data in a single resource and to aid the design and discovery of more practical MOFs by valorizing their synthesis information.

Most chemistry literature is published as unstructured text, which makes manual database creation cumbersome, time-consuming, and error prone. To address this problem, Swain and Cole developed ChemDataExtractor (CDE) to automate the extraction of chemical data from research articles and patents via text mining.^34^ To date, CDE has been deployed to automatically assemble databases of magnetic materials,^35,36^ battery materials,^37^ UV/vis absorption spectra,^38^ hydrogen storage and synthesis applications,^39^ and nanomaterial synthesis.^40−42^ While CDE has been used to text mine both organic and inorganic chemistry literatures, it has yet to be applied to MOFs, possibly due to challenges presented by the diverse nature of their building blocks and complex synthesis techniques. To the best of our knowledge, Park et al.’s text mining software was the first work which enlisted text mining to scrape MOF-related data such as pore volume and surface area.^43^ More recently, Luo et al.^44^ developed an automatic data mining tool using the CoRE MOF database,^45^ alongside the web-scraping tool Puppeteer (https://pptr.dev) to text mine 6099 journal articles. These were then analyzed using ChemicalTagger software^46^ to extract metal sources, linker(s), solvent(s), additives, synthesis time, and temperature. A further recent submission from Park et al. data mined 46,701 MOFs to extract synthesis information from 28,565 papers using a joint ML/rule-based algorithm.^47^

The CSD MOF subset contains comprehensive structural information about MOFs; however, the data related to their synthesis is scarce and inconsistent. Here, we text-mined the CSD MOF subset and developed rule-based MOF compound name and property parsers within CDE to automatically generate a database of MOF synthesis data, i.e., the DigiMOF database, to facilitate digital transformation of MOFs’ synthesis protocols. We envisage that DigiMOF will allow next-generation high-throughput screening and ML approaches to take more circumspective consideration of the synthesis information. These new features will allow MOF scientists to rapidly search for MOFs associated with specific precursors, topologies, organic linkers, and synthesis routes, offering a platform which facilitates screening and identification of sustainable and scalable materials. For each MOF compound, its corresponding DOI is also included in the database so users can access the publication where it was first reported. We highly encourage users of DigiMOF to build upon this foundational work and integrate additional MOF property extraction capabilities into the adapted CDE to expand or tailor the database according to their own research requirements.

## 2 Property Identification and Parsing

The principal challenge in developing text mining parsers is to identify key MOF properties for data extraction. Initially, we conducted an extensive review of the existing literature to select properties that are most indicative of MOF scalability and ease of synthesis. Given the widespread interest in MOF chemistry, it is somewhat surprising that only a few MOF technoeconomic assessments (TEA), with a focus on production, have been carried out. For example, DeSantis et al.^48^ demonstrated that switching from traditional solvothermal synthesis techniques to more novel, less solvent-intensive pathways such as aqueous or mechanochemical routes could reduce MOF production costs by 34–83%. Increasing the MOF yield by a factor of 30% had a negligible impact on production costs in comparison to using a less solvent-intensive pathway. In another study, Luo et al.^49^ compared traditional solvothermal synthesis with an aqueous pathway to produce UiO-66-NH_2_ and found that omitting solvents from the synthesis of this MOF resulted in an 84% reduction in production cost. The key properties that influenced the production cost were solvents, organic linkers, and inorganic MOF precursors.

Following these findings, we focused on constructing parsers to extract information on four key MOF synthesis properties: solvents, inorganic and organic precursors, and synthesis methods. We also constructed a parser to extract MOF topologies, as the description of topology aids mechanical stability predictions, critical for the pelletization and industrial application of MOFs.^50^ Finally, integration with the CSD Python API also allowed information such as the tested temperature, article DOI, and publication year to be merged with the parser-extracted records. The CSD Python API was also used to extract the chemical names that corresponded to each MOF refcode in the 3D MOF subset for linker matching.

## 3 Methods: Automatic Generation of the DigiMOF Database

**Figure 1:**
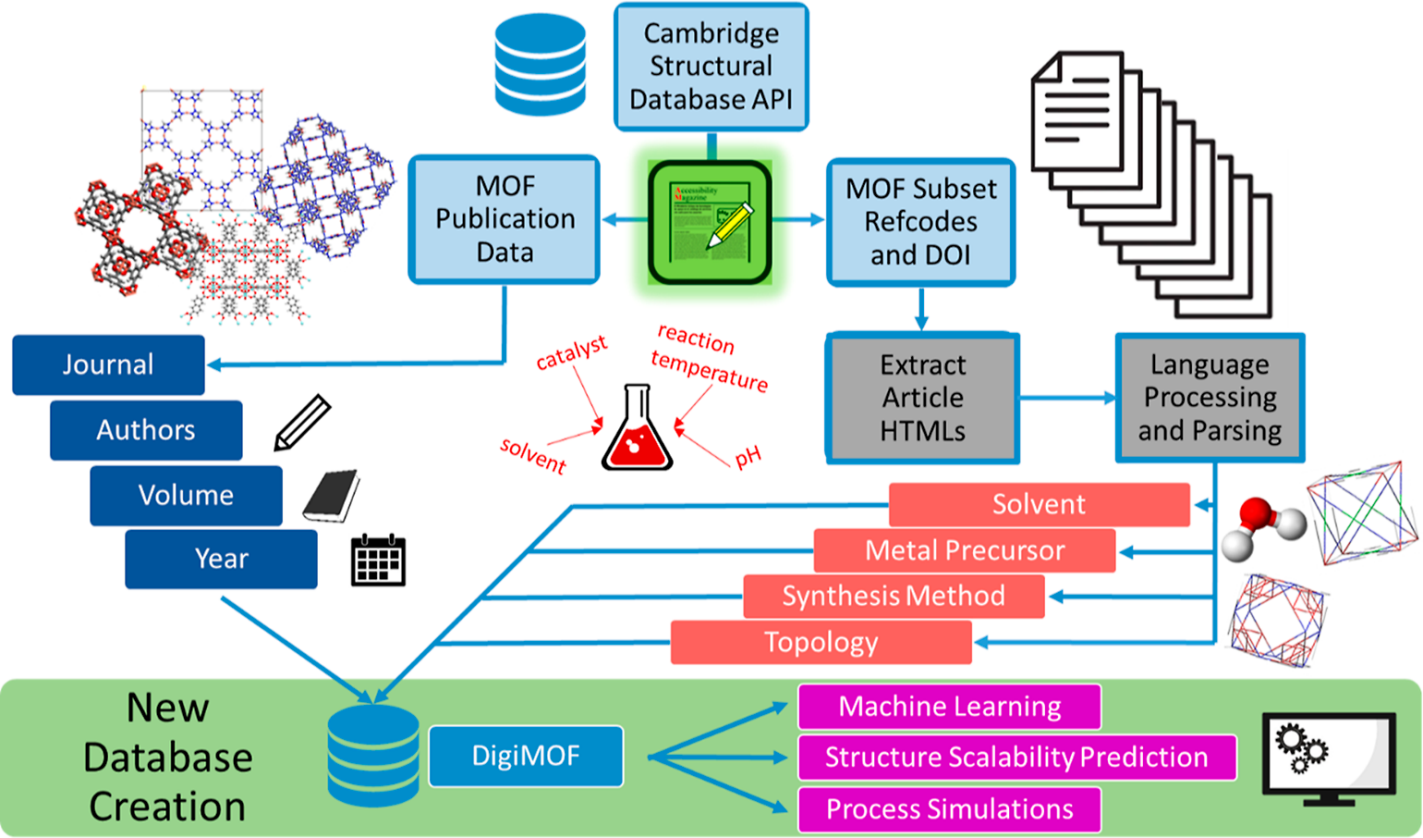
Flow diagram to visualize the integration of CDE into a data-driven MOF synthesis plan: from article retrieval to text mining, computational screening, and materials discovery.

The key motivation for adapting the CDE tool to text mine MOF literature was to better integrate MOF synthesis protocols, TEA considerations, and computational screening approaches into a tight feedback loop to enable more efficient MOF materials development. Figure 1 demonstrates how the DigiMOF database and the adapted CDE parsers can be integrated into a data-driven pipeline for MOF design and discovery.

We also developed a MOF-specific approach in conjunction with the CDE web scraper: DOIs associated with the CSD MOF subset were extracted using the CSD API and used to automatically download the associated articles in HTML format using the CDE web scraping script for the corresponding journal. After download, text-mined MOF synthesis data was automatically extracted from each HTML file and stored in our database in JSON format. This data can then be used for further TEA studies and integrated with other physicochemical properties obtained from either simulations or experiments to generate rich data sets for further processing.

Note that a user can create new and personalized databases for text mining by modifying the provided CDE web scraping script to obtain any collection of online files saved into HTML format, i.e., patents, webpages, and journal articles, from other sources.

### 3.1 Natural Language Processing

To identify specific MOF properties using CDE-based classes and variables, we created customized parsers which use part-of-speech (POS) taggers and chemical entity recognizers. These parsers contain specific regular expressions for the identification of MOF compound names. The natural language processing (NLP) pipeline in CDE first identifies a sentence, which is then tokenized into individual words and punctuation known as tokens.^34^ These tokens are marked up by POS tagging to reflect their syntactical functions, such as a noun, a verb, a chemical mention, and an adjective.^34^ Entity recognition of the chemical species allows relationships to be extracted and merged with their corresponding compounds by interdependency resolution.^34^ Our rule-based parsers used Python regular expressions as well as CDE parsing elements and were tailored to extract specific properties. We generated parsing rules to identify MOF names, synthesis methods, inorganic precursors, linker names, and MOF topology abbreviations, as well as created exclusion lists to exclude words which were frequently misidentified as these variables. The use of regular expressions and parsing elements, as shown in Table S1, was crucial to improving performance.

**Figure 2:**
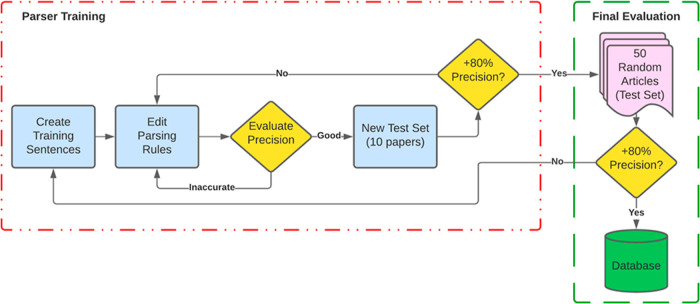
Iterative flow chart depicting the process through which the parsers were refined before database application.

The process of building and refining the parsers is shown in Figure 2 following a similar process used by Huang and Cole.^51^ First, basic parser functionality was achieved on individual sentences by successfully extracting the MOF compound name and corresponding property. The parsers were then tested on a series of sets containing 10 random papers and continuously refined until they achieved a precision above 80% on one test set. The last step of the process was evaluating parser performance on a final set of 50 randomly selected papers from the CSD.

### 3.2 Technical Validation

This text mining software was evaluated for reproducibility on a randomly selected array of “unseen” text, distinct from the training set used to refine the NLP parsers, to ensure the parser performance achieved on a limited training set can be consistently replicated for high-throughput application. The three performance metrics used in evaluation are precision, recall, and F-score, which can be calculated using eqs 1–3, respectively. True positives (TP) correspond to data extracted and identified correctly. False positives (FP) correspond to data which are incorrectly identified as a match. False negatives (FN) are relevant data which should be extracted but have not been identified.
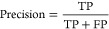
1
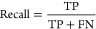
2
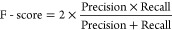
3

Precision is the fraction of correctly extracted data, recall is the fraction of available data extracted, and F-score represents the harmonic mean of recall and precision. For the estimation of precision and recall, 50 MOF articles were randomly selected as the test set from a collection of over 700 articles retrieved by the web scraper from the CSD: the selected articles can be found in the Supporting Information. For each extracted record, a value of 1 was assigned if both the MOF compound name and the corresponding property (e.g., synthesis method, linker, etc.) were correctly matched, or a value of 0 if the compound name or the property were incorrectly matched. The number of total relationships was manually extracted from the same 50 journal articles and compared with the records in the auto-generated database to calculate recall and precision.

In practice, there is often a trade-off between the precision and recall of a text mining algorithm. The development and implementation of rule-based parsers prioritize high precision, which reduces the overall recall as the parser is less capable of extracting values from many variations in sentence structure. More lenient parsing rules increase the overall number of records extracted and therefore improve recall, but they also show a reduction in specificity, which reduces precision. Generally, high precision should be given precedence over recall; low recall is acceptable provided that a large enough data set is used to compensate for a lower proportion of the available data being extracted. Examples of the compound records from this work and previous projects using CDE are shown in Table S2. We found it extremely challenging to accommodate the considerable diversity of sentence structures observed in MOF literature without compromising the precision of the parsers. When maximizing precision, extracting common and unambiguous sentences observed in MOF literature was prioritized, although it was expected that lower recall would be obtained compared to previous iterations of CDE. Figure S1 summarizes the overall performance of our parsers compared to previous CDE projects and the MOF text mining tool from Park et al.^43^ The overall precision for our parsers was 77%, which we deemed satisfactory, as values approaching 80% are generally considered sufficient for data-driven materials discovery via current text mining techniques.^51^ A breakdown of individual parser results for the synthesis route, topology, linkers, and metal precursors can be found in Table S3.

### 3.3 Parser Training

During parser training, precision was substantially improved by employing exclusion lists to filter out frequently observed misidentifications. The addition of common abbreviations, names, and exclusion list items for metal precursors, linkers, MOFs, and topologies to the regular expressions helped to improve both precision and recall. As MOF terminology and literature are dynamic and rapidly evolving, it is crucial that continued adaptations be made to this tool to improve its performance. With this idea in mind, we have made the software open source with the aim of using open collaboration to add abbreviations or names to the exclusion lists and compound regular expressions, which will allow the tool to evolve and improve over time.

**Figure 3:**
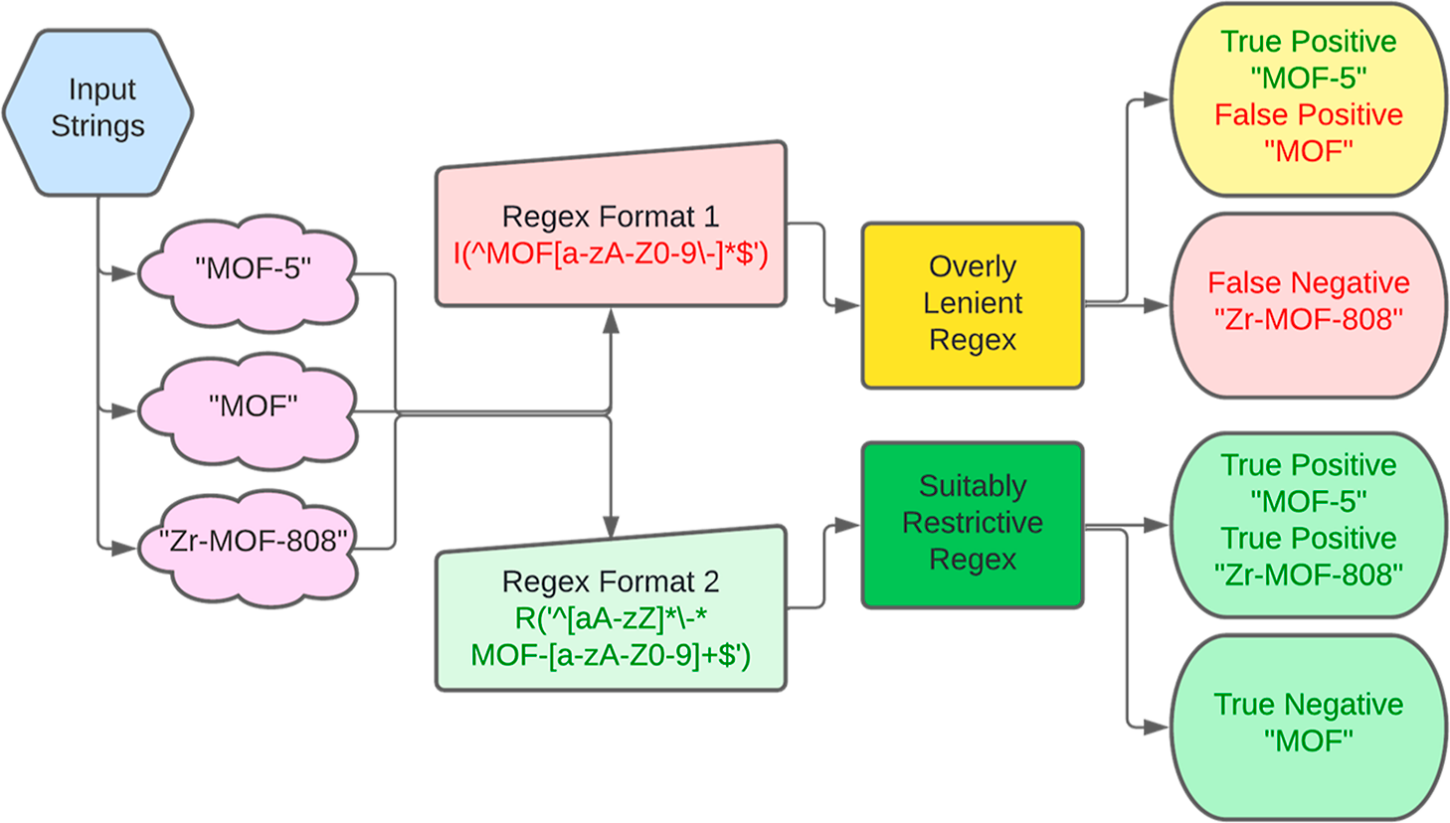
Flow chart displaying possible outcomes when fed an input string for high-throughput MOF name parsing.

Figure 3 shows the process for the selection of regular expressions that can be incorporated into CDE. Here, we demonstrate how regular expressions (regex) may be developed iteratively to achieve more TPs and eliminate FPs and negatives. Table S4 contains examples of simplified regex used in the creation of the DigiMOF database. The actual regex which have been integrated into the MOF version of CDE are available on the associated GitHub (https://github.com/peymanzmoghadam/DigiMOF-database-master-main.git) in the chemical entity mention (CEM) and precursor parser files.

It is often preferable to use multiple regular expressions to accommodate different formats of the same variable. Attempting to accommodate too many types of matches into a single expression can increase the number of FPs, as demonstrated by expression number 4 in Table S4 which is the lenient regular expression for common linker abbreviations. To accommodate a wider variety of sentence structures to help recognize MOF names, an exclusion list was integrated into the regular expression rules to exclude FPs, as with expression 9 in Table S4. Regular expressions within the context of exclusion listing are further detailed in the Supporting Information in Table S5.

### 3.4 Obtaining Metal, Topology, and Linker Data

After parsing was complete, to obtain further, more detailed information surrounding the metal elements contained with each MOF, we used a high-throughput approach that involved obtaining the relevant crystallographic information files (CIFs) for use in the MOFid software suite.^52^ Each CIF was entered into the program where it was then deconstructed, and the metals present in the MOF were extracted. For topological representations of these structures, we used the Julia-based CrystalNets^53^ program to automatically assign network topologies to all CIFs. This enabled the comparison of algorithmically assigned values from these software packages with the text-mined data for verification purposes.

**Figure 4:**
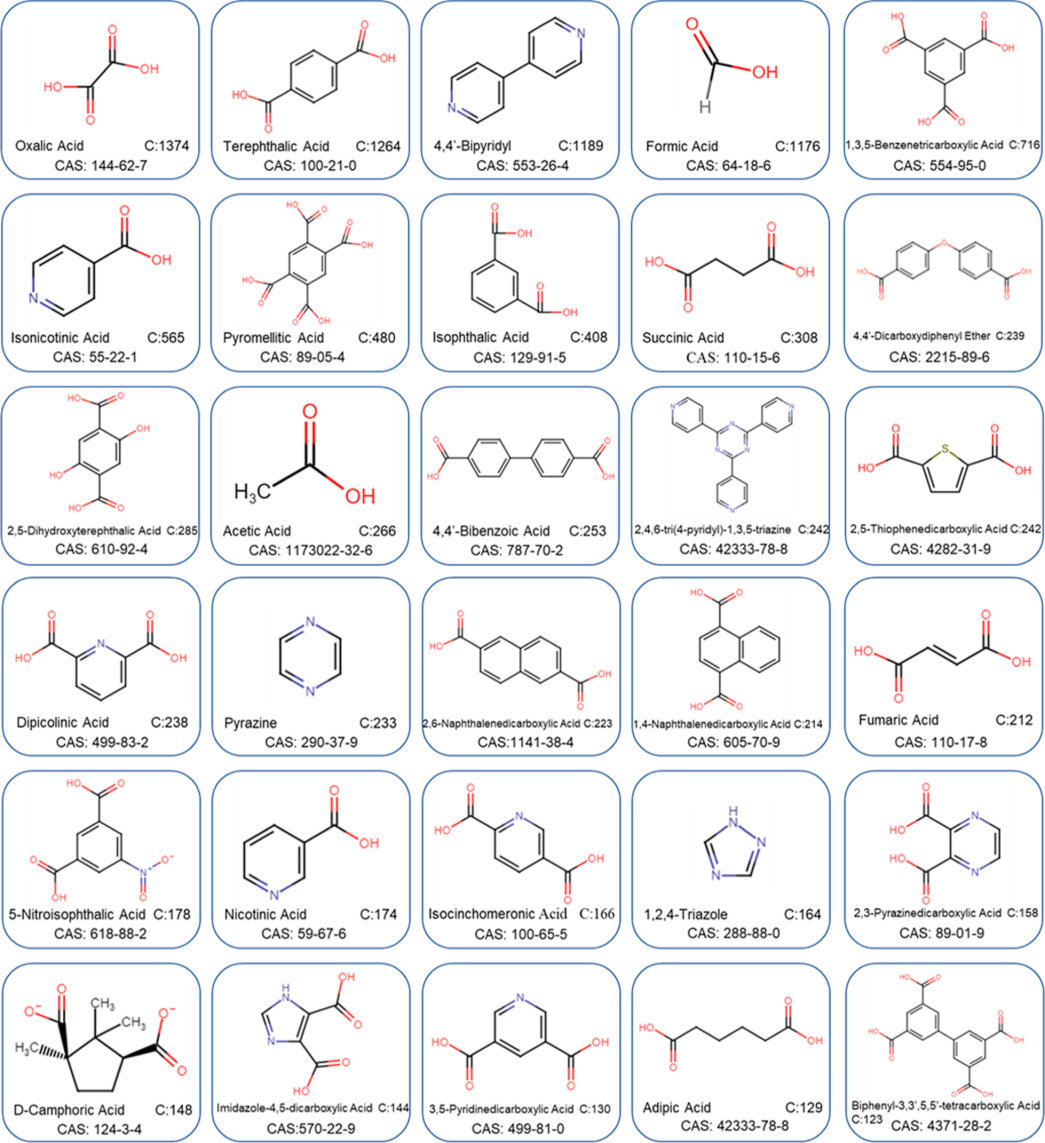
Collection of the top 30 organic linkers obtained via text-mining the CSD MOF subset chemical names. Hit counts (C) and CAS numbers are included for each linker.

Obtaining linker information proved to be more challenging. We created “rules” in the CSD Python API to extract linker names which enabled the simplification of CSD’s long text-based chemical names into distinct repeating units. For example, the chemical name for SAHYIK within the CSD is “*catena-(tris*(μ_4_-1,4-*Benzenedicarboxylato)-(*μ_4_*-oxo)-tetra-zinc octakis*(*dimethylformamide) chlorobenzene clathrate)*”. These names were initially treated by extracting the metal names, in this case zinc, and adding them to the list of metals for each structure. Then, the remaining text is split based upon the names which succeed μ, indicating that there are repeating units; the remaining non-chemical items such as “*catena-*” and “*tris*” are also discarded here. These repeating units are then transformed to match the chemical names found in the list provided by TCI Chemicals^54^ for common MOF linkers. For this first entry, e.g., “1,4-benzenedicarboxylato” is modified to “1,4-benzenedicarboxylate”, which can also be represented by its alias terephthalic acid and is then matched to the TCI Chemicals list. The second μ corresponds to the string “oxo”, which is discarded as it refers to the repeating oxygen molecules in the zinc oxide node. Anything that succeeds the metal in the chemical name and is separated by a space is removed and retained for further processing as possible solvents used in the synthesis. Figure 4 shows the outcome of this process for the 30 most frequently extracted records taken from a list of 149 unique chemical names and matched after both a manual and an automatic transformation process were performed. The matching list, which includes linker synonyms and chemical prices, can be found in the Supporting Information TCI_Chemicals (XLS) document.

### 3.5 Geometric Properties

By analyzing the text-mined data, correlations between different MOF topologies and structural properties were unveiled by determining a complete set of geometric properties and investigating the patterns which emerged from known and unknown relationships. The largest cavity diameter (LCD), pore limiting diameter (PLD), accessible surface area (ASA), frameworks density, the presence of open metal sites, and void fraction of all 3D MOFs in the subset were calculated using Zeo++ software^55^ to quantitatively characterize their structural properties. A probe radius of 1.86 Å, corresponding to the kinetic radius of N_2,_ was applied for ASA calculations. The results of these calculations can be found in the DigiMOF3DSubset (CSV) document of the Supporting Information.

## 4 Results and Discussion

We note that for a MOF compound name and the corresponding property relationship to be entered into the DigiMOF database, both the MOF compound name and property had to be recognized by the parsers. Overall, 15,501 MOF compound name and property relationships with over 52,680 associated properties were extracted from the CSD MOF subset which contains 43,281 unique MOF publications and over 100,000 MOFs. Table 1 displays the total number of each type of synthesis property associated with MOFs, in addition to the total number of unique properties of each type. The full list of MOF names and their relevant properties can be found in the Supporting Information.

**Table 1:** Total Number of Extracted Properties and the Number of Unique Properties for Each MOF Property in the DigiMOF Database

property	total extracted	total unique properties extracted
MOF compound names	15,501	
synthesis route	9705	8
Solvents	1211	81
Topologies	6680	154
Linkers	24,116	10,690
metals including ions	10,968	1803
metals excluding ions and element names	5163	1476

The DigiMOF database contains a MOF compound name and corresponding topology, organic linkers, metal precursors, synthesis methods, or solvent for approximately 15% of structures within the CSD MOF subset. One important factor to consider is that not every publication discusses all of these properties. If a compound is labeled as “1” or “2” without a specifier such as “compound”, “complex”, or “MOF”, then the parsers will not associate the label with anything and so cannot extract a property relationship. We must also note that full access to every article within the CSD was not possible, either due to the location in which the article was published or that the corresponding papers were written in languages other than English. An extended discussion on how the parsers function is located in the Database Overview and Performance section of the Supporting Information. In the following sections, we summarize our key findings after text mining the CSD MOF subset.

To enrich the database of 10,696 3D structures extracted with CDE, we also gathered additional information using alternative computational methods as detailed in Section 3.4. Table 2 shows a breakdown of the parameters we extracted and calculated to supplement the text-mined data set. A total of 24,784 3D MOFs were admitted to the calculation stages, where the constituent metal was identified for 23,832 structures, and either an RCSR or EPINET topology was assigned for 13,816 3D structures.

**Table 2:** Total Number of Extracted Properties and Number of Unique Properties for Structures in the 3D MOF Subset

property	total extracted	total unique properties extracted
MOF compound names	24,784	
Topologies	13,816	460
Linkers	15,901	129
elemental metals	23,832	716
LCD and PLD	22,104	
Density	24,587	
open metal sites	763	
geometric properties	>6474	

Here, we note that despite obtaining 10,690 unique linker names in the text mining stage for journal articles, once we take the more uniform CSD chemical names and match synonymous chemicals together, we collected information for at least one linker type for 40% of materials that have suitable chemical names for the matching process. The complete data for linker names, metals, and topologies can be found in the Supporting Information DigiMOF3D subset (CSV).

## 5 Data Analysis

### 5.1 Synthesis Methods

**Figure 5:**
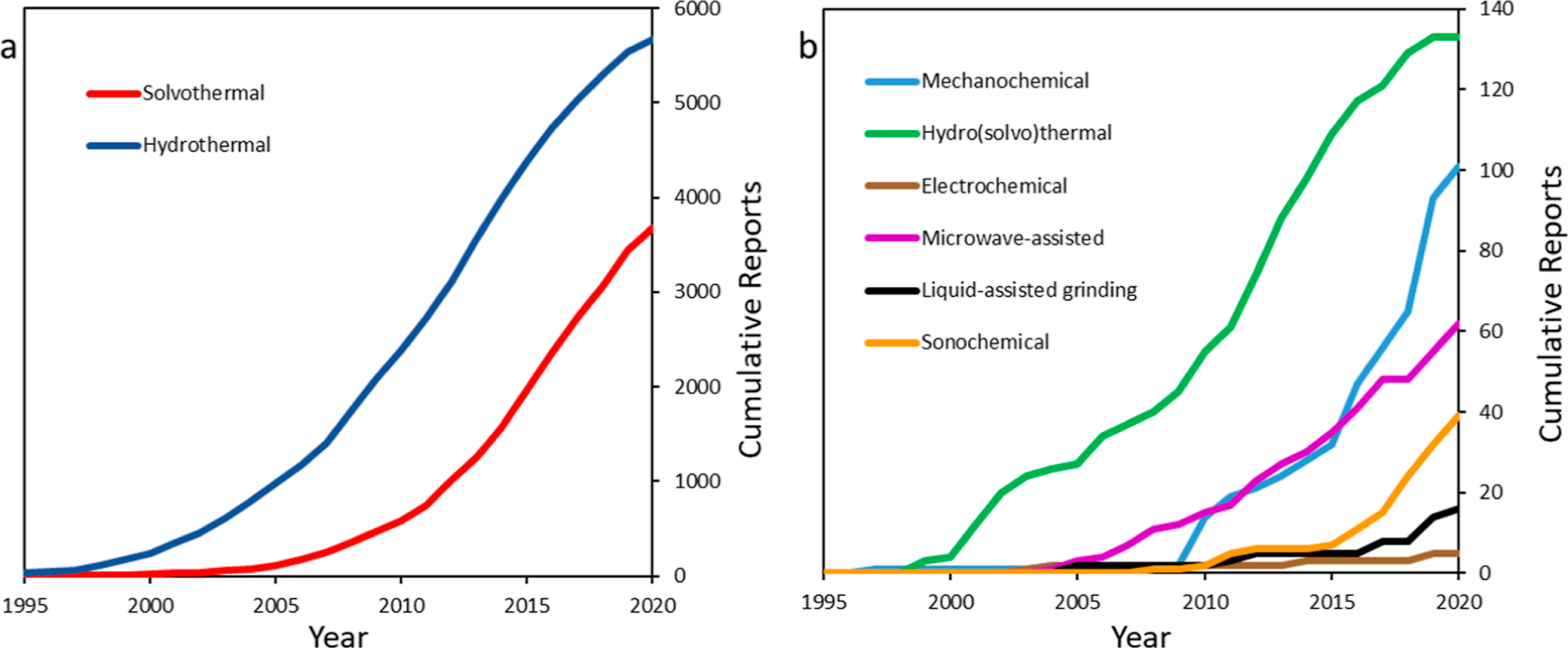
(a) Cumulative sum of the two main MOF synthesis methods from 1995 to 2020. (b) Cumulative sum of alternative and emerging synthesis methods showing periods where these techniques were first introduced for MOF synthesis.

When analyzing the data for synthesis methods, we first investigated how synthesis methods have changed over time. A total of 9705 synthesis route records were extracted from 43,281 papers. Figure 5 shows the cumulative sum of records extracted for various types of synthesis routes from 1995 to 2020. Solvothermal synthesis in the context of MOFs generally refers to the use of one or more organic solvents such as DMF and methanol at high temperatures. Hydro(solvo)thermal synthesis generally refers to reactions where water is employed as a part of a solvent mixture. Hydrothermal synthesis refers to reactions where water is the primary solvent and is itself a type of solvothermal synthesis. A significant result was the extraction of more hydrothermal (5,677) synthesis methods than solvothermal (3,672). This is surprising as the most common laboratory-scale MOF synthesis routes are solvothermal; however, many papers do not explicitly name this as their synthesis route but instead imply it by mentioning the use of solvents and high temperatures in the Methods Section. These implicit synthesis routes could be easily deduced by a reader but are challenging to extract using rule-based NLP algorithms which are looking for a specifier word such as “solvothermal”. Figure S7a also shows that hydrothermal synthesis was the most common alternative/low-solvent synthesis route extracted by the parsers.

We also note that the majority of synthesis route records are from articles published in the last 10 years; this reflects the rapidly increasing interest and investment in MOF compounds and in alternatives to the solvothermal synthesis method. In fact, 6033 (62.2%) of the total synthesis route records may be classified as alternatives to solvothermal synthesis, which reflects greater interest in developing alternative synthesis routes, particularly when considering that high solvent-use is inhibiting MOF scalability. Rapid increases can be observed for more novel synthesis routes, with an overwhelming majority of solvent-free synthesis papers published after 2010 (76% microwave-assisted, 95% sonochemical, 86% mechanochemical, and 88% liquid-assisted grinding). There is also likely to be some cross-over between these methods, as liquid-assisted grinding and sonochemical methods are themselves subsets of mechanochemical methods and may be used in various combinations for MOF synthesis. This trend of utilizing greener synthesis methods is also reflected in innovative MOF commercialization efforts such as the ton-scale water-based processes that BASF has developed^56^ and the mechanochemical process from MOF Technologies.^57^

The DigiMOF database allows users to search for potentially scalable MOFs via the synthesis method to discover MOFs that can be more easily synthesized and tested with the equipment and resources available to them. In the future, an alternative web search query method of database assembly could be used in place of the CSD reference code method to assemble a corpus using queries such as “solvent-free MOF synthesis” or “mechanochemical MOF synthesis”, expanding the database to include more MOFs that can be produced using alternative synthesis methods and novel synthesis techniques for MOFs already logged in the database with more conventional synthesis routes. The synthesis method parser should be continually updated to allow it to parse novel synthesis methods and procedures, as and when they become more prominent in MOF literature and may be extended to parse for post-synthetic methods such as linker substitution.

### 5.2 Topology

**Figure 6:**
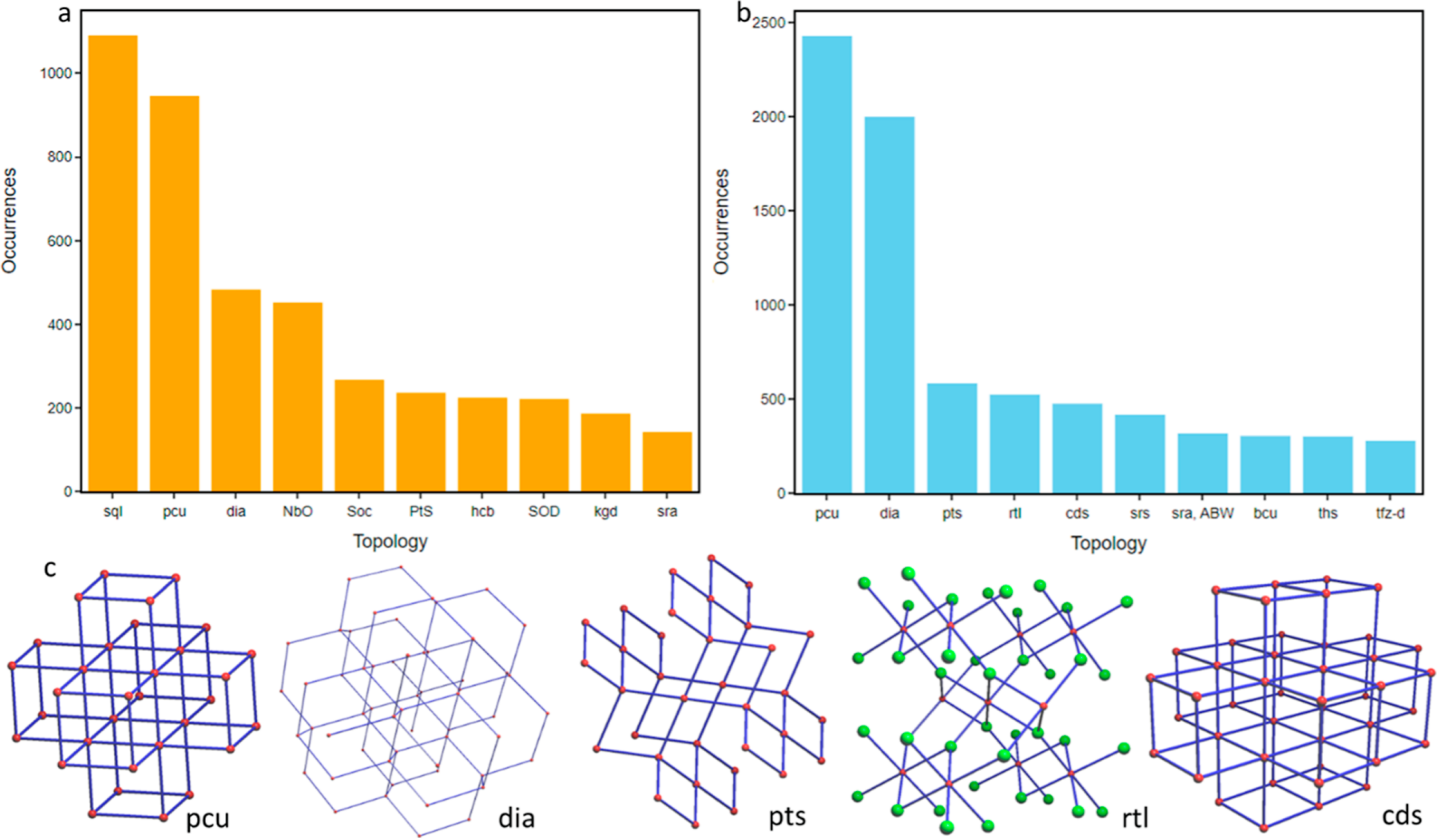
Histograms of topological types extracted from the CSD MOF subset using (a) ChemDataExtractor (CDE) (b) CrystalNets in 3D structures. (c) Top five most common 3D topologies: **pcu**, **dia**, **pts**, **rtl**, and **cds**.

Topological characterization of MOFs is important as it can constrain key structural properties such as pore shape, size, and chemistry, and it is directly related to mechanical stability.^50^Figure 6a. shows the distribution of topologies identified in the CSD MOF subset: we extracted 112 unique topologies across a total of 6680 results. The most frequently occurring topology was **pcu** with 946 hits, followed by **sql** and **dia** with 822 and 482 counts, respectively. In some publications, the parsers picked up variations of certain topologies, e.g., **sql**, **44-sql**, **(4,4)-sql**, and **(44)-sql** as separate entries. From the top ten topologies shown in Figure 6a, **sql**, **hcb**, and **kgd** are 2-periodic, and the remaining seven exhibit 3-periodic frameworks. The Supporting Information provides a full list of MOF names and topologies identified. We also performed topological characterization of the 3D MOF subset using CrystalNets^53^ and achieved a return of 55.8% across 460 unique topologies. We note here that the CrystalNets calculations allowed for the extraction of topological types that matched the EPINET^58^ database, whereas our text-mining approach was specifically developed to seek out RCSR^59^-type topologies. Figure 6b shows the occurrence of the top ten topological nets with **pcu** as the most frequently occurring topology, followed solely by 3D representations in **dia**, **pts**, **rtl**, and **cds** rounding out the top five. Figure 6c shows examples of commonly occurring 3D underlying nets. An additional outcome of this study was that 2375 structures in the 3D MOF subset were built from two or more interpenetrating nets. We anticipate that this topological characterization of MOFs will also guide future efforts to identify mechanically stable MOFs.

**Figure 7:**
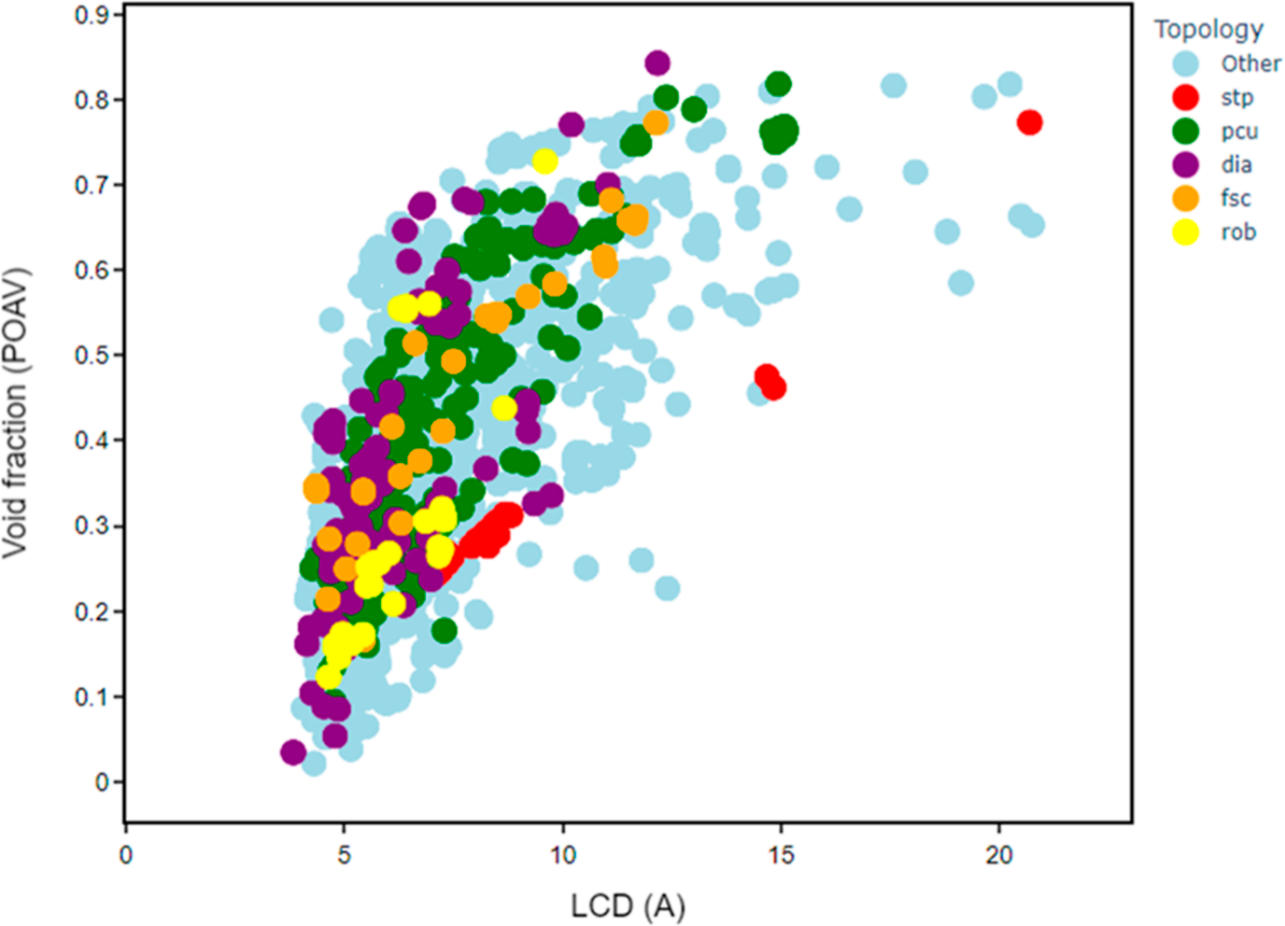
Comparison of different topologies in the structure space for LCD as a function of void fraction for ∼2200 porous MOFs. There are 241 structures with **pcu** topology (green); 170 **dia** (purple), 41 **stp** (red), 33 **rob** (yellow), and 32 **fsc** (orange) structures. All other structures are shown in pale blue.

We used the topological data to investigate the topology–structure relationships for certain geometric properties. Figure 7 shows the different regions that are occupied by a selection of five topological types. For some representations, there does not appear to be any restriction on the types of pores that can be formed with a wide variety of void fractions seen for **pcu** and **dia**. Both representations span a range of void fractions between 0 and 0.85 across and the LCD range of 3.7–15 Å. On the contrary, there are some slightly more distinct linear patterns between the LCD and the void fraction for other representations, which are particularly noticeable for **stp** and **rob**. The former shows a distinct linear pattern within the region of 5 to 10 Å and 0.2 to 0.35 void fraction and displays a similar linearity into the 15 to 20 Å range.

### 5.3 Solvent

Dimethylformamide (DMF) is the most frequently extracted solvent, representing 469 of the 1211 extracted solvents. Water is the second most frequently extracted solvent with 186 counts for which 127 were paired with hydrothermal synthesis routes. The remainder of the water solvent records were merged with solvothermal or hydro(solvo)thermal synthesis routes, which could reflect the common use of solvent mixtures containing multiple reagents such as DMF, water, and ethanol. The parser does not have the capability to extract lists or mixtures of solvents unless they appear consecutively in a string without whitespace, e.g., “DMF/H_2_O”. The additional top hits for solvent extraction can be seen in Figure S7c.

The presence of organic solvents such as DMF, DMA, ethanol, and acetonitrile demonstrates that despite increased research into alternative synthetic pathways, many existing synthetic procedures are still reliant on organic solvents and failure to eliminate large volumes of such solvents in MOF synthesis is one of the largest barriers to MOF commercialization. It should be noted that while the CSD includes solvent information, most of these records are missing from the database. These parsers offer the ability to search for MOF synthesis routes associated with a given solvent, thereby allowing researchers to limit screening to hydrothermal synthesis or to solvothermal synthesis techniques with cheaper, less toxic, or more readily recoverable solvents.

### 5.4 Organic Linkers

Histograms in Figure S7d show that carboxylate-type linkers were the most frequently extracted type of organic linkers, with over 432 associated records. Specific carboxylate linkers, e.g., benzene dicarboxylate acid (BDC), were not extracted more frequently because these linkers are more generically referred to as carboxylate or dicarboxylate without specification of the exact structure. Other challenges with NLP parsing of MOF linkers in the literature were inconsistencies in linker abbreviations and naming conventions. For example, “bpy” and “bipy” are used to denote specific bipyridine-type linkers such as 2,2-bipyridine and 4,4-bipyridine.^60,61^ While researchers may be referring to specific linkers when using these abbreviations, these labels are not consistently used to refer to any one distinct structure. Records for “bpy” and “bipy” were merged as “bipy” to denote generic bipyridine-type linkers. Following data transformation where instances of “4,4-bipyridine”, “4,4-bipy”, and “4,4-bpy” were merged as “4,4-bipy”; 273 records were associated with “4,4-bipy” and 267 with “bipy” representing the 2nd and 3rd most extracted linkers, respectively. Similar transformations were conducted for 2,2-byripidine linkers with 109 records. Carboxylate (H3BTC, BDC, carboxylate, dicarboxylate) and pyridyl-type linkers (4,4-bipy, 2,2-bipy, bipy, bpe, and bpp) were the most dominant linker types extracted by the parsers. Other notable linkers included imidazole-type bridging ligands such as “bimb” (phenylenebis(methylene)bis(1*H*-imidazole)). “H2L” was the 4th most extracted linker with 251 associated records. This does not refer to a specific chemical structure; instead, it is a generic label used within the MOF literature to refer to a number of organic linkers.^62^ This means that the linker chemical formulae may be explicitly named in one part of the text and then simply be referred to as “L”, posing considerable challenges for NLP parsing. In some instances, researchers do not elaborate on the chemical formula of the linker within any part of the text and use a generic L-type notation or refer to the general structure (e.g., carboxylate). The usage of generic labels and general compound class names may reflect increased trends toward more complex and functionalized linkers in MOF synthesis, which may make consistent identification and naming of these structures more challenging.^63^ The chemical diversity of MOF linkers is an important factor, particularly when considering the application of ML on these data sets.^64^

**Figure 8:**
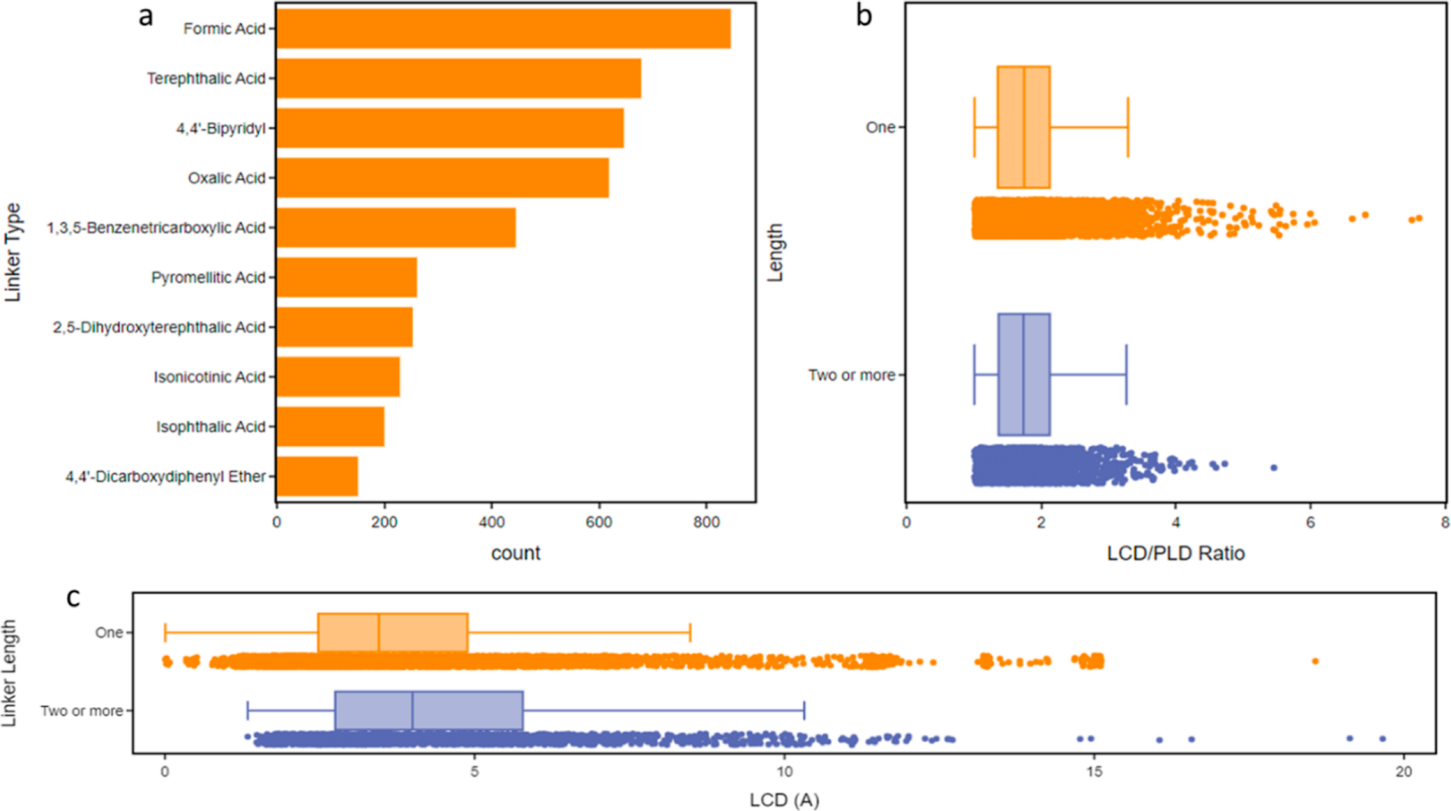
(a) Histogram showing the most commonly occurring single linkers found in the 3D MOF subset for non-zero LCD values. (b) Box and whisker plot of linker length versus the LCD/PLD ratio across a sample of ∼8000 MOFs. (c) Box and whisker plot of linker types against LCD for a sample of linkers with one (orange) and two or more (blue) blocks.

To combat this ambiguity, we developed a new approach to text extracting MOF linker names using the chemical names found in the CSD, as these are available for over 99% of all deposited structures. The result of this text mining required some manual intervention as CSD chemicals can have different naming protocols; for example, one might find 1,3,5-benzenetricarboxylate or the synonymous benzene-1,3,5-tricarboxylate both used within this data set. There are in fact tens of examples of similar synonymous chemical names being used across the 149 linker names we used as our match list. Overall, this new method had a significantly higher accuracy given the strict designations for similar linker molecules. For example, the distinction between 4,4′-bipyridine and 2,2′-bipyridine, when compared to the CDE text mining results, avoids the need to note “generic bipyridine-type linkers” and enables deeper analysis of similarly named but chirally different molecules. Figure 8a shows the frequency at which a linker type was reported for structures that contained reference to only a single linker but also had a non-zero cavity diameter. This data was then used to separate linkers depending on their length, which was determined by the number of consecutive blocks, e.g., number of benzene or pyridyl rings, into categories of 1 or 2+ blocks. The difference between the linker length and their respective MOF LCD ranges is shown in Figure 8b. We note here that the longer 2+ block linkers have a larger LCD range from 1.3 to 12.3 Å, whereas shorter one-block linkers span a slightly smaller range of LCD values from just above 0 to 8.5 Å. Interestingly, despite the mean LCD following the pattern of increasing with linker length, there are several one-length linker structures that far exceed the average LCD of MOFs built with two or greater length linkers. Once the linkers had been categorized with respect to their length, it was possible to investigate the pore morphology, as shown in Figure 8c, a box and whisker plot of linker length against the LCD/PLD ratio. The results here suggest that shorter linkers with one block can generate structures with a wide range of LCD/PLD ratios, whereas longer linkers containing 2+ blocks generate structures on lower ranges of LCD/PLD ratios of <2.5: a finding which is dominantly due to larger PLD values in these structures.

### 5.5 Metal Precursor

The choice of metal precursors is also important for MOF synthesis; certain metal clusters such as metal oxides can provide cost-effective and flexible MOF production routes as well as control over structural topology and shape. Our parser extracted many metal precursors in the form of a metal element, ion name, or symbol: this is shown in Figure S7e. Zinc-based precursors were most frequently extracted, with “Zn(NO_3_)_2_·6H_2_O” representing 365 of the merged records. Zinc salts represented three of the most extracted metal precursors, accounting for 36% of the 1481 records. This is unsurprising given the prevalence and popularity of zinc-based MOFs; however, the absence of zirconium salts from the top 10 metal precursors is unexpected. One reason for the lack of zirconium salts is that papers discuss zirconium precursors as “Zr”, as can be seen by 212 hits in the database for “Zr”, shown in Figure S3. Additionally, compared to zinc and copper-based MOFs, Zr-based MOFs were not widely produced until after 2012.^65^ The second most frequently extracted metal salt was “Cd(NO_3_)_2_·4H_2_O” with 177 merged records, followed by nitrate salts of Zn, Co, and Cu. The ability to cross-reference MOF structures with their metal precursors from proven synthesis procedures will allow MOF scientists to rapidly screen structures for criteria such as metal nodes or precursors associated with desirable properties, greater material abundances, and lower costs. Searching by metal precursors will also provide valuable insight into MOF building blocks in cases where records include MOF names which are not directly based on the MOF structure or formula.

**Figure 9:**
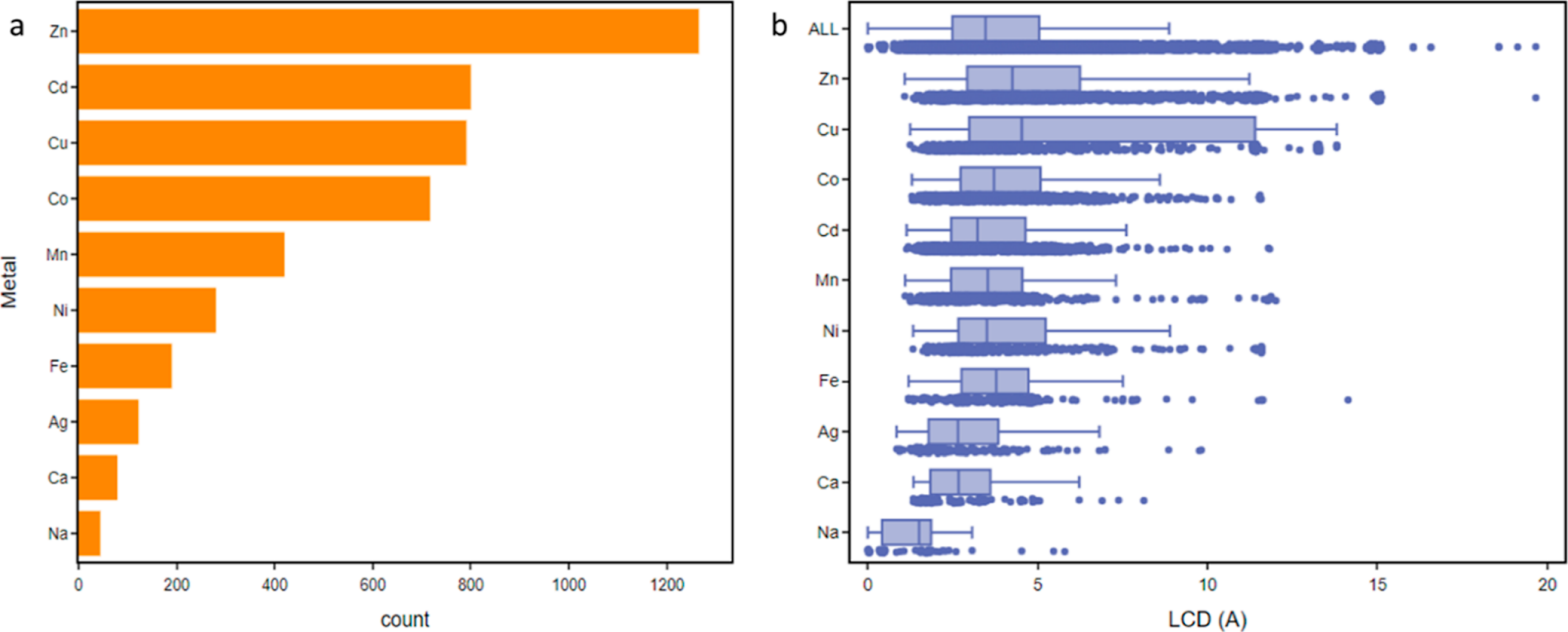
(a) Histogram of the most frequently occurring single metals found in the 3D MOF subset. (b) Comparison of the constituent metals against the LCD of structures.

Figure 9a shows the most frequently occurring single metal types in the MOF subset as identified using MOFid.^52^Figure 9b shows the relationship between metal types and the typical LCD values expected for each MOF containing that metal. The most common metal, Zn, contains over 1200 entries in the database, for which 752 or 60% can be considered porous such that they have an LCD which exceeds the probe diameter of 3.7 Å. For Co and Cd, this ratio decreases to 51 and 41%, respectively. The lowest proportion of porous MOFs from the metals can be found for structures containing Na, where only 34% of entries have LCDs greater than 3.7 Å. Na-containing MOFs have the lowest mean LCD of all metals at 1.5 Å, whereas Cu-MOFs have the highest at 4.6 Å, with the average LCD across all metals sitting at 3.5 Å.

### 5.6 Temperature

The CSD database contains temperature entries for almost all deposited structures when DOI records were extracted from the CSD Python API, it was also possible to extract corresponding temperature records without error. The results of these extractions, which have been rounded to the nearest whole degree Kelvin, can be seen in Figure S7f—it is important to note that these values are not the synthesis temperatures of the materials but are of the variable-temperature crystallographic studies. These are the temperatures used in post-synthesis investigations at which the results of certain experimental procedures in each manuscript have been reported, specific to each material. This data does not guarantee the stability of MOFs at these temperatures. Typically, an experimental structure is tested and reported at or around room temperature, explaining the spike in records at 293 K. It is also common that a Cryostream or other device is used to cool a sample for low-temperature crystallographic testing. We would recommend the introduction of more useful temperature data fields, such as activation temperature, destabilization temperature, or solvent/synthesis/reaction temperature, alongside the existing crystallographic study temperatures.

### 5.7 Building Blocks and Topology

The underlying networks of the extracted MOF structures can be investigated using insight gained from the data presented in Figure S4. There are 4972 linker hits for which there was a corresponding topology and a further 1424 results for metal clusters. Taking into consideration the top five most frequently parsed linkers and metal precursors from Figure S7d,e respectively, we can deduce the top five topologies for each MOF building block. These results are represented in a clustered column graph, Figure S4. Furthermore, additional data obtained via CrystalNets^53^ has offered insight into the topological configuration of 3D MOFs in the DigiMOF database, with a return rate of ∼55%. A filter can be applied to this data set to select all matched linker types for a given topology.

The top linker type extracted using CDE, [“carboxylate”] corresponded to a total of 100 topologies, the most frequent being **sql** (12), and **pcu** (12). These two topological types emerged as the most frequent for almost all investigated linkers and metal clusters, an unsurprising result considering the high frequency of these two representations across the whole study. These are two of the simplest underlying structure representations, which may explain their abundance; more complex structures are less likely to have topology reports due to potential errors, and additionally, it is common to report the most simplified underlying net even where a more complex representation exists. For the 3D data set, the highest linker type [“oxalic acid”] corresponded to a total of 66 unique topologies, with the most frequent being **dia** (84), followed by **pcu** (50).

In 2014, a study by Cai et al. investigated the crystal structures of derivatives of HKUST-1, which notes that for H-BTC (the 5th most common linker type), the predicted topological type is **tbo**; however, variations in the functionalization of this same linker can give rise to a preference for **fmj** connectivity using the same building blocks.^66^

Perhaps more interesting than the results for linkers is that of metal clusters; typically, linkers are connected only at each edge, although in some less common cases (e.g., where linkers consist of porphyrins and derivatives), there can be a higher number of connections. Depending on the coordination of certain metal clusters, it can be impossible to achieve some topological types, making the choice of the metal cluster more restrictive than the choice of the linker; a significant influence on the potential underlying network of a crystal structure. From these metal cluster results, we can deduce that transition-metal nitrate structures form some of the simplest underlying nets with **sql**, **pcu**, **dia**, and **kgd** being frequently reported in synthesis papers. This variety of 2 and 3 dimensional, and 4-connected, 6-connected, and 6 plus 3-connected clusters suggests flexibility in the coordination number of these transition-metal building blocks.

Further to this point, it is worth noting the influence of temperature on the dimensionality of MOF structures. Reaction temperature has been found to have a remarkable influence on the formation and structure of MOFs, especially toward the control of topology.^67^ Increasing the hydro/solvothermal reaction temperature has the potential to increase the coordination number of the central metal ion.^68^ Anderson et al. suggested that a temperature-dependent quantity such as free energy, which would have a notable influence toward the topological selectivity of MOF synthesis, should be considered in MOF synthetic accessibility predictions.^69^

### 5.8 Cost Analysis

**Figure 10:**
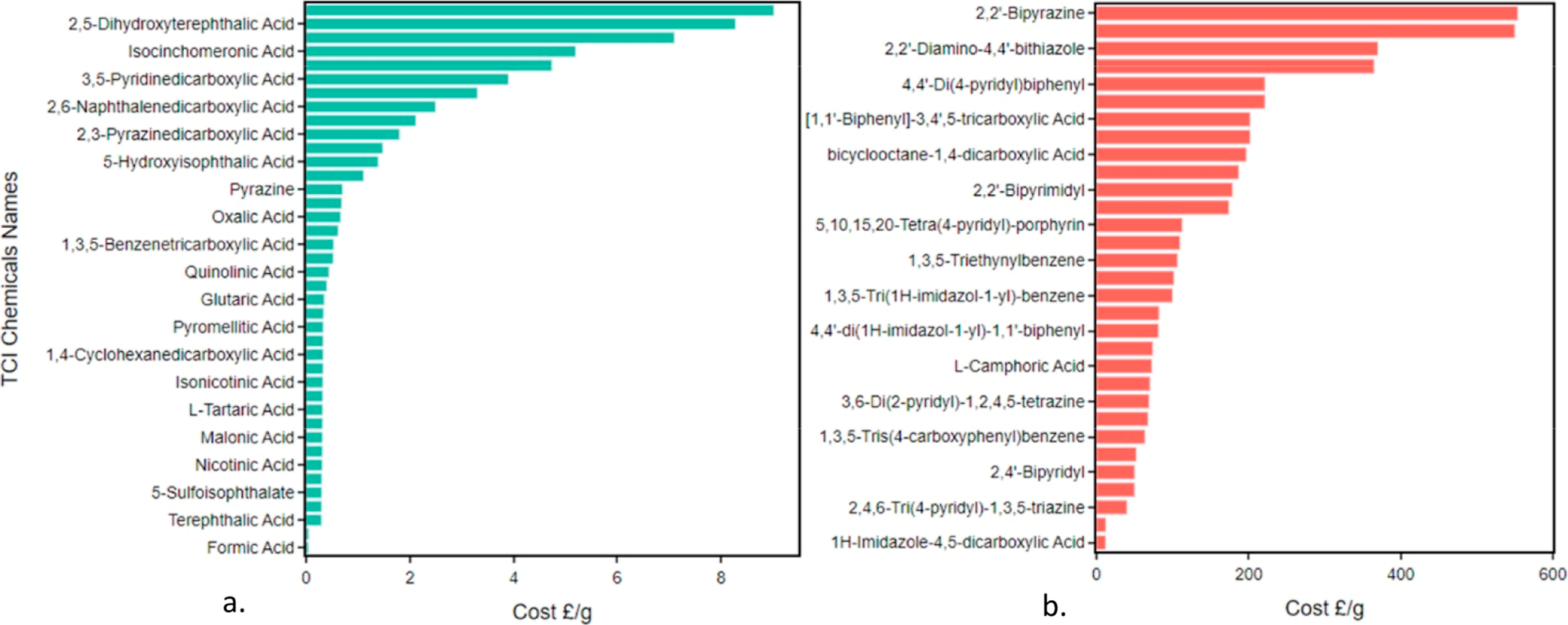
Bar charts showing the cost per gram of organic linkers as determined by averaging the available quantities. A selection of the most prevalent linker types was chosen from the DigiMOF database for (a) low-cost and (b) high-cost linkers. Prices obtained from TCI Chemicals.^54^

As a result of improving the accuracy in linker designation from Section 5.4, and from the use of a matching list modified from the publicly available TCI Chemical list, it was possible to add an approximate linker cost analysis to our data set, given the availability of pricing data for these chemicals.^54^ We took the TCI Chemicals list and added several other commonly used organic materials, followed by the inclusion of live online prices, these costs are typically for quantities of 99%+ purity precursor chemicals. Due to the inclusion of additional listings, it was necessary to obtain some missing cost values from Sigma-Aldrich to get a complete list of approximate linker “raw chemical” costs.^70^ The available quantities varied between all linker types, and so the prices in this list were determined by taking into consideration all of the possible prices and finding the mean cost per gram. Figure 10 shows the results of the linker cost analysis on some of the most prevalent linkers detailed in Section 3.4. As the structures obtained from the CSD MOF subset are experimental, we expected to see most of the structures containing lower cost linkers for the simple reason that they would be more economical to produce. While the range of linker costs across the chemical list spans £0.05 to £830 per gram, out of the top 45 linkers, 40 of them had a cost per gram under £10, as can be seen in Figure 10a. This sample of linkers in the “low-cost” range spans a total of 6643 structures. Figure 10b also shows a total of 33 linkers that exceed a cost of £10 per gram, although they make up a much smaller proportion of the total structures that have been identified as linkers in this study.

The results of this cost analysis can be used to select specific linker types for techno-economic assessment in conjunction with limiting solvent quantities, finding optimal reaction temperatures, selecting suitable catalysts, and selecting low-cost metals. The cost per mole of each linker type can also be found in the Supporting Information document, TCI_Chemicals (XLS).

## 6 Conclusions and Future Directions

To the best of our knowledge, the DigiMOF database is the first automatically generated database of MOF synthesis properties using ChemDataExtractor to text-mine 43,281 MOF publications. After an iterative training process, the parsers yielded an overall precision of 77% to extract 52,680 associated MOF synthesis properties. This initial text-mined data was supplemented with additional data mined from the CSD MOF subset, which enabled the identification of linker types and their corresponding costs. DigiMOF will allow researchers to search for key properties related to implementing large-scale MOF production, e.g., synthesis routes and solvents, organic linkers, metal precursors, structure topology, constituent metals, and linker cost. We envisage DigiMOF as an invaluable tool to both MOF scientists conducting high-throughput computational screening and experimentalists evaluating MOF properties empirically. The software and the parsers developed here are open-source to allow researchers to update our regular expressions as new compounds emerge, ensuring these algorithms can continue to identify new MOF-property relationships. With minimal additional effort, researchers can employ the modified CDE scripts to generate their own database; with more focused search queries to study alternative MOF production pathways by making very basic alterations to the parsers. The ability to cross-reference and merge data using DOIs allows researchers to readily merge or expand this database to include other properties, which pique their interest.

DigiMOF is primarily focused on the production of MOF compounds but also includes basic geometric properties to offer an additional level of insight. Additional parsers can be developed to extract properties related to scalability and synthesis, such as the reaction temperature, space-time yield, heat of adsorption, reaction time, and regeneration time—all essential parameters for enhancing MOF synthesis pathways. We also recommend that future MOF synthesis publications contain specifically formatted tables of key information as an appendix to the article, presented in a way that is friendly to text mining algorithms to enable the scraping of data using a high-throughput screening approach, improving both the precision and recall of any chemical journal parser. By improving the precision and recall of structure property parsing beyond the levels we see today, there is the potential to enable an accurate and reliable database of synthesis data to be created in the public domain that can be continually and accurately updated following new publications.

We envisage that this work will lay the foundation for enabling digital manufacturing of MOFs and facilitate the identification of commercially viable MOF production pathways. With over 15,000 unique MOF records, this data can be used to further assess the viability of alternative MOF synthesis routes and to drive further techno-economic assessment, life-cycle assessment, and experimental validation work. DigiMOF could therefore help to reduce the overdependence within the MOF community on unsustainable synthesis routes, which currently precludes the application of these structures in decarbonization technologies that motivate many contemporary MOF research proposals. With thousands of entries for each parameter parsed in this study, DigiMOF augments MOF scientists’ expertise, allowing them to design more efficient MOF discovery pathways and advance the synthesis of these fascinating materials.

## Supporting Information Available

DigiMOF3DSubset (XLSX)
TCI_Chemicals (XLSX)
Parsing elements to identify MOF names and the corresponding topology, solvent, synthesis route, organic linker, and/or metal precursor; compound records from previous versions of CDE; MOF CDE parser performance compared with previous versions of CDE; summary of the performance of each individual parser; simplified MOF CDE regular expression (Regex) examples; examples of organic linker exclusion list item regular expression development; proportion of synthesis methods present in the MOF database; histogram displaying the 25 most extracted strings marked up as metal precursors; clustered columns reflecting the top five topological allocations to the top five linker types and metal clusters; top 20 topologies versus the LCD/PLD ratio in descending order of frequency for structures with PLD>0.55; box and whisker plots of linker length against the LCD/PLD ratio for all porous MOFs; and histograms showing the most common MOF properties extracted in the DigiMOF database (PDF)

## Notes

5D visualization platform on Wiz: all of the graphs presented in this paper can be reproduced online at https://wiz.shef.ac.uk/ when the Wiz-friendly file format is used. Furthermore, visitors to the site can explore the entire data set interactively, with all the variables plotted on each of the five axes according to the user’s interest. MOFs can be searched for and filtered either by name or by property or by selecting them from the graph.^71^
