# Supplementary material for: DigiMOF: A Database of Metal–Organic Framework Synthesis Information Generated via Text Mining
Source: Chem Mater. 2023 May 18;35(11):4510–24. doi: 10.1021/acs.chemmater.3c00788 (PMC10269341; doi:10.1021/acs.chemmater.3c00788)
Supplement: Supplementary file 3 — cm3c00788_si_003.pdf [file cm3c00788_si_003.pdf]

# DigiMOF: A Database of Metal-Organic Framework Synthesis Information Generated via Text Mining

*Lawson T. Glasby[a], Kristian Gubsch[a], Rosalee Bence[a], Rama Oktavian[a], Kesler Isoko[a], Seyed Mohamad Moosavi[c], Joan L. Cordiner[a], Jason C. Cole[d], and Peyman Z. Moghadam[a,b]**

[a] Department of Chemical and Biological Engineering, The University of Sheffield, Sheffield, S1 3JD, United Kingdom.

[b] Department of Chemical Engineering, University College London, London, WC1E 7JE, United Kingdom.

[c] Chemical Engineering & Applied Chemistry, University of Toronto, Toronto, Ontario, M5S 3E5, Canada.

[d] Cambridge Crystallographic Data Centre, Cambridge, CB2 1EZ, United Kingdom

*Email: p.moghadam@ucl.ac.uk

# Supporting Information

## Article Retrieval

Article retrieval is achieved by using DOIs to automatically download articles from journal websites. Two methods may be used to retrieve article DOIs when assembling a corpus of MOF articles using CDE. For the first method, a web scraping script developed in the most recent version of CDE can be used to send a search query to Elsevier and the Royal Society of Chemistry to extract the DOIs which are then used to download the article in the form of a HTML file. We also developed a second method which involves retrieving MOF reference codes and their associated DOIs from the Cambridge Structural Database (CSD) using the CSD Python API. Both methods produce a CSV file which stores the DOIs of the articles to be downloaded. Here, we used the CSD Python API as utilising search queries was found to significantly increase the time required for web scraping. We wrote a Python script which calls the Selenium webdriver to navigate to the article webpage and the PyAutoGui library to save the articles as HTML files. After running the web scraping script, to get access to the publications, a window appears where the user must sign

into their DOI account via their institution's website. The scraper will then automatically copy and paste article DOIs from the CSV file where they are stored, prior to downloading them. The web scraping script can download approximately three articles per minute. We recommend researchers use high-performance computing clusters to assemble corpuses that contain thousands of articles to avoid a bottleneck in the pipeline.

## Database Overview and Performance

As the data mined for the DigiMOF database consisted of text-text relationships, in contrast to the text-numerical records from previously conducted text mining studies, there were considerably more linguistic and syntactical variations in the reporting of the properties of interest compared to previous projects.

**Table S.1** Parsing elements used to create the rule-based grammars to identify MOF names and corresponding topology, solvent, synthesis route, organic linker, and/or metal precursor[1].

| Element | Description | Element | Description |
|---|---|---|---|
| R(Regex) | Match text with regular expression | T(Tag) | Match tags |
| W (Word) | Match case-insensitive token text | I(Iword) | Match case-insensitive token text |
| Any | Match any single token | H(Hide) | Ignore the matched tokens |

| | | | |
|---|---|---|---|
| Not | Match only if not followed by some text | FollowedBy | Match only if followed by some text |
| ZeroOrMore | Match zero or more of the expressions | OneOrMore | Match on or more of the expressions |
| Optional | Match if it exists | SkipTo | Skips to the next occurrence of text |

**Table S.2** Examples of compound records which were extracted in this project and in previous chemistry text mining projects. Previous projects enlisting CDE mined text-numerical data, whereas this project mined qualitative text-text relationships. Note that for the example for this database a real record was used and in this instance no topology or solvent was associated with the MOF compound by the parser. This is typical as it is rare for all 5 properties to be found in one compound record. It has been attempted to represent records from previous projects as faithfully as possible but the exact format of the scraped records is not always available in the source material.

**Table S.2** Examples of compound records from previous versions of CDE, the previous attempt to text mine MOF data and this work.

| Source | Project | Compound | Properties | | |
|---|---|---|---|---|---|
| **This Work** | *CDE MOF Synthesis Routes* | **Names:** MOF-5 | **Linker:** $H_2BDC$ | **Metal_precursor:** Zinc(II) terephthalate | **Synthesis_route:** solvothermal |

| | | | | | | | |
|---|---|---|---|---|---|---|---|
| **Huang and Cole**[1] | *CDE Battery Materials Database* | **Names:**<br>Li1.5Co02 | **Current_value:**<br>16 | **Current_units:**<br>mAg-1 | **Cycle_value:**<br>25 | **Cycle_units:**<br>cycles | |
| **Court and Cole**[2] | *CDE Neel and Curie Temperatures* | **Names:**<br>BiFeO3 | **Temp_type:**<br>Curie | **Temp_value:**<br>647 | **Temp_units:**<br>K | | |
| **Park et al.**[3] | *MOF Surface Area and Pore volume* | **Names:**<br>MOF-210 | **Surface_type:**<br>BET | **Surface_value:**<br>6240 | **Surface_units:**<br>$m^2/g$ | **Pore_volume_value:**<br>3.6 | **Pore_volume_units:**<br>$cm^3/g$ |

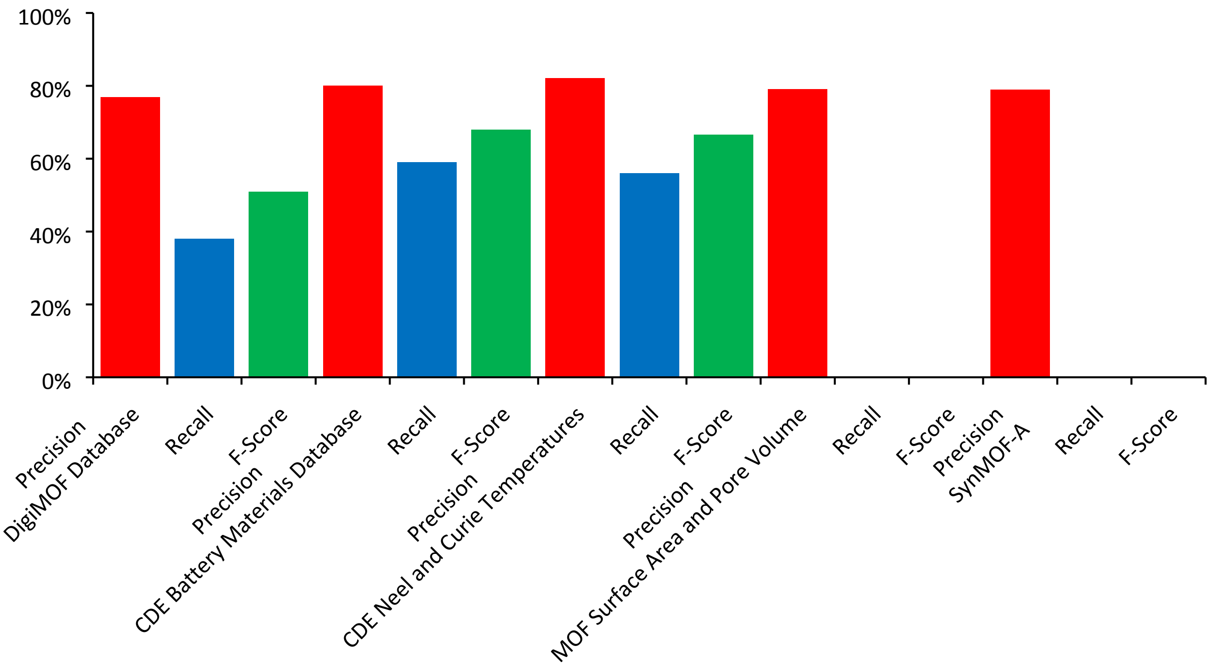


**Figure S.1** MOF CDE parser performance compared with previous versions of CDE[1,2] and the work from Park et al.[3] MOF text mining tool. Performance of individual parsers and detailed methodology for calculation of these metrics is available in the supporting information, **Table S.3**.

The machine-learning assisted version of CDE enlisted in the Neel and Curie Temperature database achieved a precision of 82% on its test-set, but this is expected to converge to 66% over time as the algorithm is trained on broader datasets[2]. Park et al.[3] reported their accuracy to be 79% but recall and F-score were not reported in their work. Luo et al.[4] also had an accuracy report of 78.9% which was referred to as consistency, this value was obtained by matching the manually extracted records in the SynMOF-M database with the automatically extracted records in the SynMOF-A database. It's also important to note that when sentences contained multiple compound names associated with other properties, our parsers could only identify the properties correctly if the MOF compound name was preceded or followed by a property without another MOF compound name separating the two. Some sentences however have multiple MOF names listed first with their corresponding properties listed second and this resulted in the erroneous association of the last MOF compound name with the first property name. Finally, a filter for MOF names was created using a regular expression to ensure that only MOF compound names were extracted into the database which further limited the entries, increasing precision.

The performance of each individual parser was also manually assessed on 50 random journal articles. For each property (synthesis routes, topologies, solvents, linkers, and metal precursors) in the database, both the precision and recall was calculated as shown in **Table S.3** below.

**Table S.3** Summary of the performance of each individual parser.

| Property | Precision | Recall | F-score |
|---|---|---|---|
| Synthesis Route | 100% | 37.7% | 54.7% |
| Topologies | 70% | 40% | 50.9% |
| Linkers | 62.9% | 35.9% | 45.7% |
| Metal Precursors | 89.4% | 40.3% | 55.6% |

For each property, the precision was calculated by manually extracting each property from all 50 papers. Following this, the values extracted by the parsers were given the value of "1" if the match was correct and a value of "0" if the match was incorrect. The total of correct extractions was then divided by the total number of identified properties (incorrect are false positives) to obtain the precision. To calculate the recall, the total of correct values was divided by the all the correct possible values (false negatives) that the parsers could have extracted from the papers.

**Table S.4** contains a list of regular expression samples which were used to parse the respective properties. These expressions were modified and refined to improve on the aforementioned recall and precision metrics of the parser techniques for the DigiMOF database.

**Table S.4** Simplified MOF CDE Regular Expression (Regex) examples.

<table>
<tr><th>Variable</th><th></th><th>CDE Regex</th><th>Evaluation</th><th>TP</th><th>FP</th><th>FN</th></tr>
<tr><td>MOF names</td><td>1.</td><td>I(^MOF[a-zA-Z0-9\-]*$’)<br>Too lenient</td><td>Rejected</td><td>“MOF-5”</td><td>“MOF”<br>“Moffat”</td><td>“Zr-MOF-808”</td></tr>
<tr><td></td><td>2.</td><td>R(‘^MOF-[a-zA-Z0-9]+$’)<br>Only matches MOF and a suffix</td><td>Accepted</td><td>“MOF-5”</td><td>-</td><td>“Zr-MOF-808”</td></tr>
<tr><td></td><td>3.</td><td>R(‘^[aA-zZ]*\-*MOF-[a-zA-Z0-9]+$’)<br>Matches MOF and a suffix with an optional prefix</td><td>Accepted</td><td>“MOF-5”<br>“Zr-MOF-808”</td><td>-</td><td>-</td></tr>
<tr><td>Common Linkers</td><td>4.</td><td>R(‘H(2|3)[A-Z]+’)<br>Too lenient</td><td>Rejected</td><td>“$H_2$”BDC”<br>“$H_2$DABCO”</td><td>“$H_2O$”</td><td>“$h_2$bdc”</td></tr>
<tr><td></td><td>5.</td><td>R(‘H(2|3)[BDCTANHFPVIMEP]+’)<br>Only matches capitalised linker abbreviations</td><td>Accepted</td><td>“$H_2$BDC”</td><td>-</td><td>“$H_2$DABCO”<br>“$h_2$bdc”</td></tr>
<tr><td></td><td>6.</td><td>I(‘H(2|3)[BDCTANHFPVIMEP]+’)<br>Case-insensitive version of 5</td><td>Accepted</td><td>“$H_2$BDC”<br>“$h_2$bdc”</td><td>-</td><td>“$H_2$DABCO”</td></tr>
<tr><td></td><td>7.</td><td>I(‘h2dabco’)<br>Case insensitive match to only these characters</td><td>Accepted</td><td>“$H_2$DABCO”</td><td>-</td><td>“$H_2$BDC”<br>“$h_2$bdc”</td></tr>
</table>

| | | | | | | |
|---|---|---|---|---|---|---|
| **Metal Precursors** | *8.* | R(‘Fe[0-9\+\-A-Za-z]*’)<br>**Too lenient** | Rejected | “Fe”<br>“$FeSO_4$” | “$Fe_3OH(H_2O)_2O[(BDC)]_3$”<br>“Feral”<br>“Ferrous” | - |
| | *9.* | R(‘Fe[0-9\+\-A-Z]+’)<br>**Matches both MOF and metal salt names. Exclusion list can be used to exclude MOF names.** | Accepted only with exclusion list item | “$FeSO_4$” | “$Fe_3OH(H_2O)_2O[(BDC)]_3$” | “Fe” |
| | *10.* | R(‘Fe\W’)<br>**Use of a word boundary only matches with the metal element formula** | Accepted | “Fe” | - | - |

**Table S.5** below demonstrates the development of regular expressions to eliminate compounds which were frequently misidentified as linkers in exclusion lists. This is not shown for metal precursors and MOF names as the principle was similar and misidentifications were much less common for these variables as their definitions included less ambiguous regular expressions. As with the regular expressions for the variable definitions, strings may be added to the exclusion list if they have false negatives but should be avoided if they have false positives as this will prevent the identification of compounds such as linkers (which is why expression 5 may be preferred to expression 2). For abbreviations or compound names which convey ambiguity or overlap between variable types, it can be advisable to use more tailored and/or case-sensitive regular expression which corresponds to a limited number of strings or to a unique string of characters (as with

expressions 5 and 6), rather than attempting to accommodate or exclusion list many strings using more general rules. These examples are simplified and the actual exclusion list regular expressions can be found in the MOF CDE on GitHub.

**Table S.5** Simplified examples of organic linker exclusion list item regular expression development. Note that compound types (such as MOF names and metal precursors) were also added to this exclusion list.

| CDE Style Exclusion List Regular Expression | | Evaluation | TP | FP | FN |
|---|---|---|---|---|---|
| *1.* | R('[A-Za-z0-9]*OH')<br>**Too lenient, exclusion lists common carboxylic acid type linker formulae** | Rejected | "$CH_3OH$"<br>"$OH^-$"<br>"OHIO" | "$C_6H_5COOH$" | "methanol"<br>"2,4-dimethyl-3-pentanol"<br>"DMF" |
| *2.* | R('A-Za-z0-9\(\)\-\,]*\W[Aa]cid')<br>**Too lenient, exclusion lists common carboxylic acid type linker names** | Rejected | "Formic acid"<br>"Acetic acid" | "Dicarboxylic acid" | "DMF" |
| *3.* | R('^[CH0-9]*OH$')<br>**Exclusion lists solvent and adsorbate alcohol formulae** | Accepted | "$CH_3OH$" | - | "methanol"<br>"2,4-dimethyl-3-pentanol"<br>"DMF" |
| *4.* | R('[A-Za-z0-9\(\)\-\,]*[Oo][Ll]')<br>**Exclusion lists solvent and adsorbate alcohol names** | Accepted | "methanol"<br>"2,4-dimethyl-3-pentanol" | - | "DMF" |
| *5.* | I('acetic\Wacid')<br>**Exclusion lists only one commonly-used MOF synthesis modulator** | Accepted | "Acetic acid" | - | "methanol"<br>"2,4-dimethyl-3-pentanol"<br>"DMF" |
| *6.* | I('dmf')<br>**Exclusion lists only one commonly-used MOF** | Accepted | "DMF" | - | "methanol" |

| | | |
|---|---|---|
| **synthesis abbreviation** | **solvent** | “2,4-dimethyl-3-pentanol” |

## Synthesis Proportionality

**Figure S.2** is a pie chart representation of the proportionality of synthesis techniques extracted over the previous 25 years of MOF synthesis, spanning the period of 1995 to 2020. The results show a significant preference for hydrothermal techniques over the next leading method. The third most commonly reported technique has a share of only 1.44% of all reported techniques, although we must note that it is anticipated this is an even smaller proportion due to the non–specific reporting of the most common techniques which is noted in many MOF synthesis papers. Despite the increasing prevalence of novel techniques, they at present likely make up significantly less than 4% of all MOF synthesis pathways.

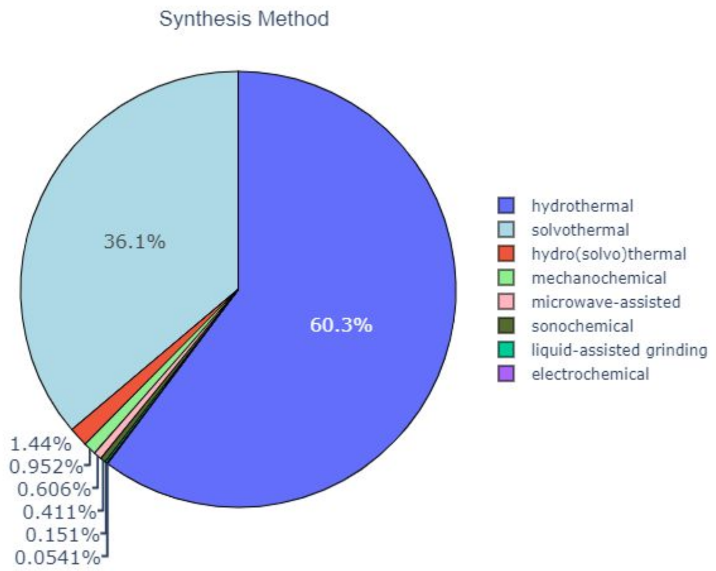


**Figure S.2** Proportion of synthesis methods present in the MOF Database.

## Data Transformation and Visualization

Following the data extraction process, the data was converted from a JSON format to a Microsoft Excel (.csv) file. While the filter was able to exclude many non-MOF names, it did miss some such as "[Co2]", "Cd-", and "Cu()" which were therefore removed using Excel's find and replace function. Additionally, the transfer of the data to Excel format led to the addition of special characters such as "Â", "â€, and "â^ž" and these were also removed. Furthermore, data that were obviously not linkers or metal precursors such as "KOH" and "NbO" were also deleted from the

database with notes made of frequent misidentifications to be added to exclusion lists. During this transformation process, synonyms were also combined such as “DMF”, “N,N-dimethylformamide”, and “dimethylformamide” to ensure that data entries were only counted once. After the data was transformed, it was combined with the data extracted from the CSD using Excel’s Power Query which combined the data based on the article download number which corresponded to the row number in the CSD thus matching the two separate data records. Figure S.3 shows the most extracted metal precursor results which were deemed not suitable to reported in the metal precursor histogram. This chart can be compared with Figure 4 e) within the main manuscript to reflect on the impact of conducting data transformation and augmentation following extraction.

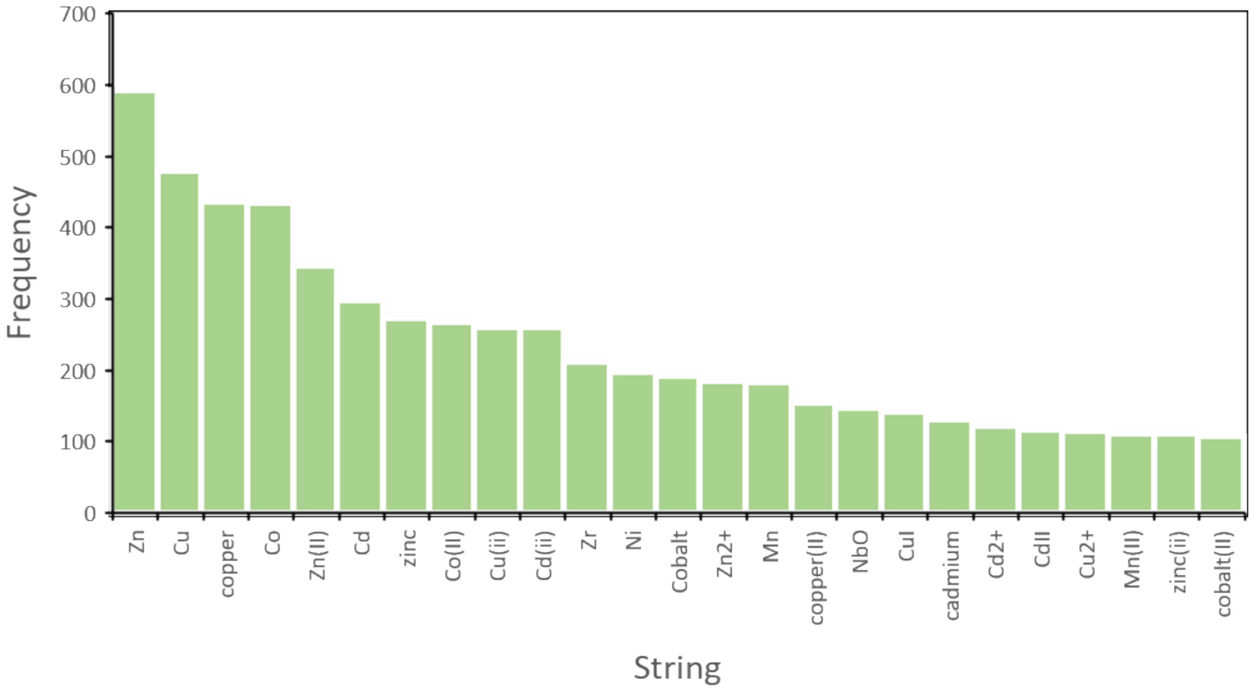


**Figure S.3** A histogram displaying the 25 most extracted strings marked up as metal precursors.

## Building Blocks and Topology

Further analysis was performed to compare the most common MOF building blocks and available topologies. The structures which most commonly reported topology and metal cluster in the experimental manuscripts were all metal nitrates, and primarily hydrated nitrates of transition metals. As for the linker types, these are primarily organic compounds which bond to metal clusters at each end of a straight chain. In **Figure S.4**, 'bipy' refers exclusively to 2,2'-bipyridine.

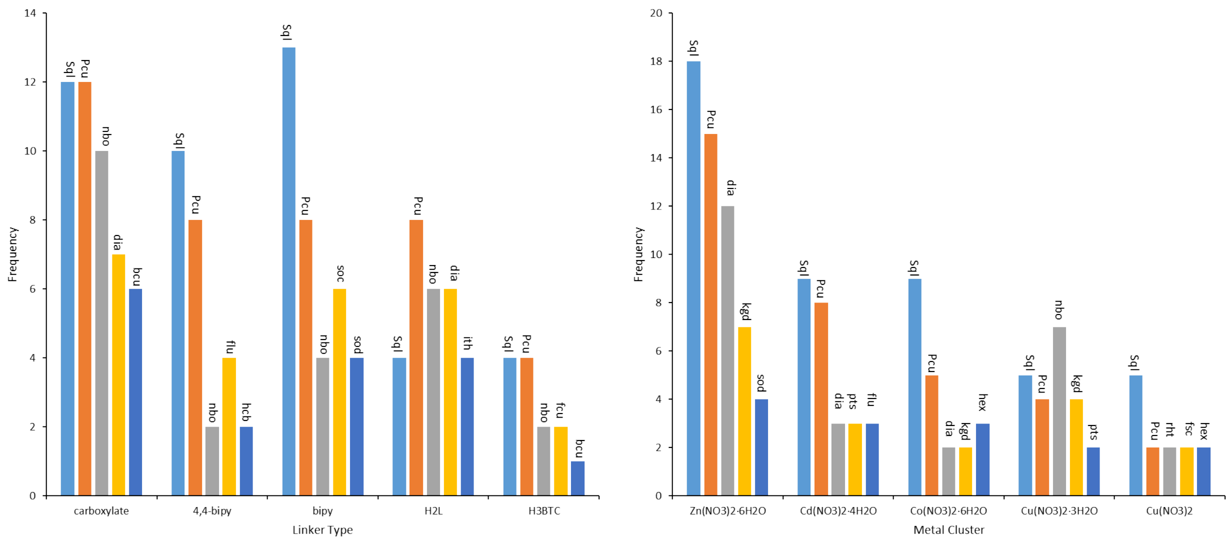


**Figure S.4** Clustered columns reflecting the top five topological allocations to **a.** the top five linker types, and **b.** the top five metal clusters.

We also investigated the ratio of LCD/PLD of the building blocks with respect to the topology. **Figure S.5** shows the top 20 topologies for porous MOFs in the 3D MOF subset against the LCD/PLD ratio across the whole range of linker types. Here we note that the diversity of the LCD/PLD ratio suggests different pore accessibility, noting that the median ratio for each topology is different. For LCD/PLD ratio close to 1, we expect to see channel type pores, whereas for larger ratios we expect larger pores and small PLD values.

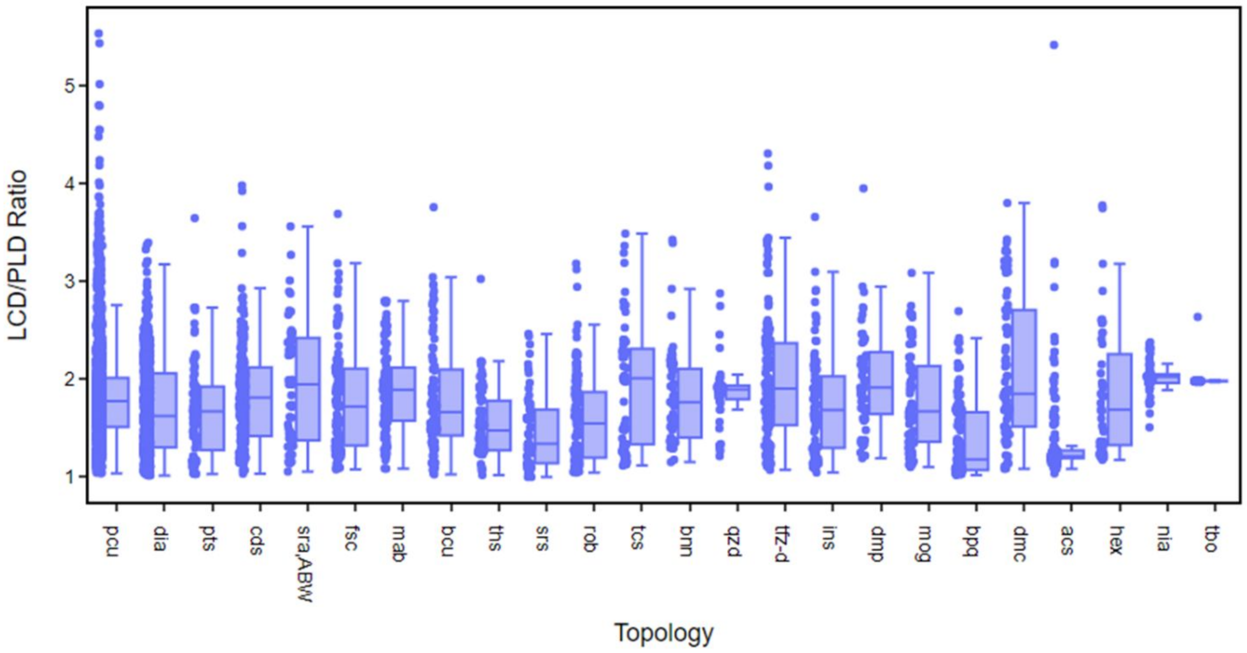


**Figure S.5** Top 20 topologies versus the LCD/PLD ratio in descending order of frequency for structures with PLD > 0.55 Å.

Lastly, **Figure S6.** shows a comparison of the linker length against the LCD/PLD ratio for the **pcu** topology. At shorter lengths, the ratio can be seen to reach a higher maximum value of 5.5, as well as a higher median value. This pattern of ratio decrease continues as the linker length increases. Given that the topology is the same across all of these structures, it would be unlikely that this change in ratio could be attributed to a structure being restricted to a single pore shape as opposed to a variety of mesopores and micropores. Whilst the pore sizes may vary, we would expect to see more uniform pore shapes for matching topologies, however given that **pcu** is one of the most basic nets, and that the topological assignment was performed using the Single Node algorithm, it

is possible that these structures do form different structures and therefore do display some variety in micro and mesopores.

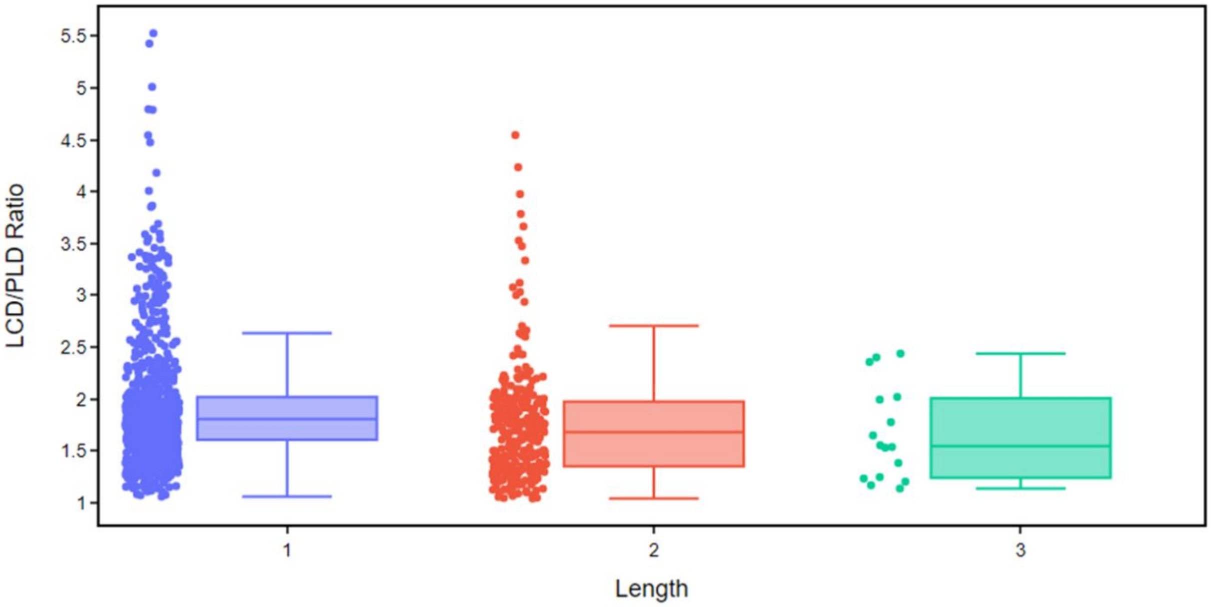


**Figure S.6** A box and whisker plot of linker length, as categorised by the number of aromatic rings, against LCD/PLD ratio for all porous MOFs which were assigned **pcu** topology.

## Text Mining Overview

An overview of the outcome of text mining can be found in **Figure S.7** where the five main parameters are listed with their properties listed in order of recurrence frequency, alongside the CSD temperature values. All data presented here were obtained using the modified ChemDataExtractor found in the associated GitHub.

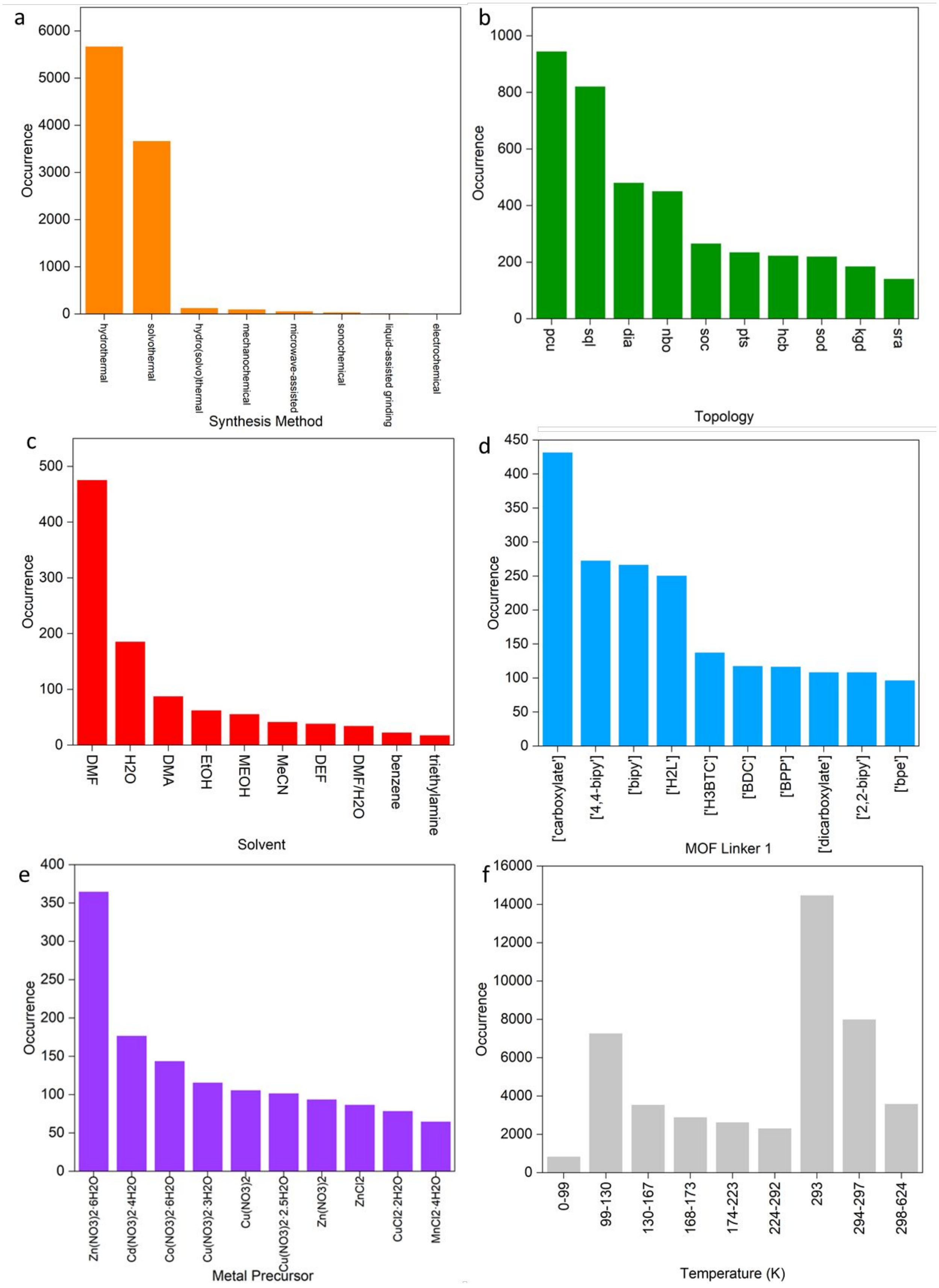

a
Occurrence
6000
5000
4000
3000
2000
1000
0
hydrothermal
solvothermal
hydro(solvo)thermal
mechanochemical
microwave-assisted
sonochemical
liquid-assisted grinding
electrochemical
Synthesis Method
b
Occurrence
1000
800
600
400
200
0
pcu
sql
dia
nbo
soc
pts
hcb
sod
kgd
sra
Topology
c
Occurrence
500
400
300
200
100
0
DMF
H2O
DMA
EtOH
MEOH
MeCN
DEF
DMF/H2O
benzene
triethylamine
Solvent
d
Occurrence
450
400
350
300
250
200
150
100
50
0
['carboxylate']
['4,4-bipy']
['bipy']
['H2L']
['H3BTC']
['BDC']
['BPP']
['dicarboxylate']
['2,2-bipy']
['bpe']
MOF Linker 1
e
Occurrence
400
350
300
250
200
150
100
50
0
Zn(NO3)2·6H2O
Cd(NO3)2·4H2O
Co(NO3)2·6H2O
Cu(NO3)2·3H2O
Cu(NO3)2
Cu(NO3)2·2.5H2O
Zn(NO3)2
ZnCl2
CuCl2·2H2O
MnCl2·4H2O
Metal Precursor
f
Occurrence
16000
14000
12000
10000
8000
6000
4000
2000
0
0-99
99-130
130-167
168-173
174-223
224-292
293
294-297
298-624
Temperature (K)

**Figure S.7**. Histograms showing the most common MOF properties extracted in the DigiMOF database. **a.** synthesis methods, **b.** topologies, **c.** solvents, **d.** organic linkers, **e.** metal precursors, and **f.** temperature.

## Parsed Articles

The test set of 50 journal articles can be found below.


(1) Hu, X.-L.; Qin, C.; Wang, X.-L.; Shao, K.-Z.; Su, Z.-M. A Luminescent Dye@MOF as a Dual-Emitting Platform for Sensing Explosives. *Chem. Commun.* 2015, *51* (99), 17521–17524. https://doi.org/10.1039/C5CC07004J.

(2) Song, C.; Hu, J.; Ling, Y.; Feng, Y.; Krishna, R.; Chen, D.; He, Y. The Accessibility of Nitrogen Sites Makes a Difference in Selective CO2 Adsorption of a Family of Isostructural Metal–Organic Frameworks. *J. Mater. Chem. A* 2015, *3* (38), 19417–19426. https://doi.org/10.1039/C5TA05481H.

(3) Yi, F.-Y.; Jiang, H.-L.; Sun, Z.-M. Linearly Bridging CO2 in a Metal–Organic Framework. *Chem. Commun.* 2015, *51* (40), 8446–8449. https://doi.org/10.1039/C5CC01244A.

(4) Ahrenholtz, S. R.; Landaverde-Alvarado, C.; Whiting, M.; Lin, S.; Slebodnick, C.; Marand, E.; Morris, A. J. Thermodynamic Study of CO2 Sorption by Polymorphic Microporous MOFs with Open Zn(II) Coordination Sites. *Inorg Chem* 2015, *54* (9), 4328–4336. https://doi.org/10.1021/ic503047y.

(5) Zhang, S.-Y.; Zhang, X.; Li, H.; Niu, Z.; Shi, W.; Cheng, P. Dual-Functionalized Metal-Organic Frameworks Constructed from Hexatopic Ligand for Selective CO2 Adsorption. *Inorg Chem* 2015, *54* (5), 2310–2314. https://doi.org/10.1021/ic502921j.

(6) Hu, Z.; Huang, G.; Lustig, W. P.; Wang, F.; Wang, H.; Teat, S. J.; Banerjee, D.; Zhang, D.; Li, J. Achieving Exceptionally High Luminescence Quantum Efficiency by Immobilizing an AIE Molecular Chromophore into a Metal–Organic Framework. *Chem. Commun.* 2015, *51* (15), 3045–3048. https://doi.org/10.1039/C4CC07642G.

(7) Paraschiv, C.; Cucos, A.; Shova, S.; Madalan, A.; Maxim, C.; Visinescu, D.; Cojocaru, B.; Parvulescu, V.; Andruh, M. New Zn(II) Coordination Polymers Constructed from Amino-Alcohols and Aromatic Dicarboxylic Acids: Synthesis, Structure, Photocatalytic Properties, and Solid-State Conversion to ZnO. *Crystal Growth & Design* 2015, *15*, 799–811. https://doi.org/10.1021/cg501604c.

(8) Yang, F.; Zheng, Q.; Chen, Z.; Ling, Y.; Liu, X.; Weng, L.; Zhou, Y. A Three-Dimensional Structure Built of Paddle-Wheel and Triazolate-Dinuclear Metal Clusters: Synthesis, Deformation and Reformation of Paddle-Wheel Unit in the Single-Crystal-to-Single-Crystal Transformation. *CrystEngComm* 2013, *15* (35), 7031–7037. https://doi.org/10.1039/C3CE40855H.

(9) Yang, Y.-Y.; Lin, Z.-J.; Liu, T.-T.; Liang, J.; Cao, R. Synthesis, Structures and Physical Properties of Mixed-Ligand Coordination Polymers Based on a V-Shaped Dicarboxylic Ligand. *CrystEngComm* 2015, *17* (6), 1381–1388. https://doi.org/10.1039/C4CE02163K.

(10) Tu, B.; Pang, Q.; Wu, D.; Song, Y.; Weng, L.-H.; Li, Q. Ordered Vacancies and Their Chemistry in Metal Organic Frameworks. *Journal of the American Chemical Society* 2014, *136*. https://doi.org/10.1021/ja5063423.

(11) Kapelewski, M. T.; Geier, S. J.; Hudson, M. R.; Stück, D.; Mason, J. A.; Nelson, J. N.; Xiao, D. J.; Hulvey, Z.; Gilmour, E.; FitzGerald, S. A.; Head-Gordon, M.; Brown, C. M.; Long, J. R. M2(m-Dobdc) (M = Mg, Mn, Fe, Co, Ni) Metal-Organic Frameworks Exhibiting Increased Charge Density and Enhanced H2 Binding at the Open Metal Sites. *J Am Chem Soc* 2014, *136* (34), 12119–12129. https://doi.org/10.1021/ja506230r.

(12) Zhao, N.; Sun, F. X.; He, H.; Jia, J.; Zhu, G. Solvent-Induced Single Crystal To Single Crystal Transformation and Complete Metal Exchange of a Pyrene-Based Metal–Organic Framework. *Crystal Growth & Design* 2014, *14*, 1738–1743. https://doi.org/10.1021/cg401887b.

(13) Patel, D. G. (Dan); Walton, I. M.; Cox, J. M.; Gleason, C. J.; Butzer, D. R.; Benedict, J. B. Photoresponsive Porous Materials: The Design and Synthesis of Photochromic Diarylethene-Based Linkers and a Metal–Organic Framework. *Chem. Commun.* 2014, *50* (20), 2653–2656. https://doi.org/10.1039/C3CC49666J.

(14) Crane, A. K.; Wong, E. Y. L.; MacLachlan, M. J. Metal–Organic Frameworks from Novel Flexible Triptycene- and Pentiptycene-Based Ligands. *CrystEngComm* 2013, *15* (45), 9811–9819. https://doi.org/10.1039/C3CE41459K.

(15) Makal, T. A.; Zhuang, W.; Zhou, H.-C. Realization of Both High Hydrogen Selectivity and Capacity in a Guest Responsive Metal–Organic Framework. *J. Mater. Chem. A* 2013, *1* (43), 13502–13509. https://doi.org/10.1039/C3TA12761C.

(16) Schoedel, A.; Boyette, W.; Wojtas, L.; Eddaoudi, M.; Zaworotko, M. J. A Family of Porous Lonsdaleite-e Networks Obtained through Pillaring of Decorated Kagomé Lattice Sheets. *J Am Chem Soc* 2013, *135* (38), 14016–14019. https://doi.org/10.1021/ja406030p.

(17) Han, L.; Xu, L.-P.; Qin, L.; Zhao, W.-N.; Yan, X.-Z.; Yu, L. Syntheses, Crystal Structures, and Physical Properties of Two Noninterpenetrated Pillar-Layered Metal–Organic Frameworks Based on N,N′-Di(4-Pyridyl)-1,4,5,8-Naphthalenetetracarboxydiimide Pillar. *Crystal Growth & Design* 2013, *13*, 4260–4267. https://doi.org/10.1021/cg400454c.

(18) Li, J.-R.; Yu, J.; Lu, W.; Sun, L.-B.; Sculley, J.; Balbuena, P. B.; Zhou, H.-C. Porous Materials with Pre-Designed Single-Molecule Traps for CO2 Selective Adsorption. *Nat Commun* 2013, *4* (1), 1538. https://doi.org/10.1038/ncomms2552.

(19) Dau, P. V.; Polanco, L. R.; Cohen, S. M. Dioxole Functionalized Metal–Organic Frameworks. *Dalton Trans.* 2013, *42* (11), 4013–4018. https://doi.org/10.1039/C3DT32588A.

(20) Qin, Y.; Feng, X.; Luo, F.; Sun, G.; Song, Y.; Tian, X.; Huang, H.; Zhu, Y.; Yuan, Z.; Luo, M.; Liu, S.; Xu, W. A Microporous Metal–Organic Framework Containing an Exceptional Four-Connecting 4264 Topology and a Combined Effect for Highly Selective Adsorption of CO2 over N2. *Dalton Trans.* 2012, *42* (1), 50–53. https://doi.org/10.1039/C2DT31905E.

(21) Zhang, J.-P.; Zhu, A.-X.; Chen, X.-M. Single-Crystal X-Ray Diffraction and Raman Spectroscopy Studies of Isobaric N2 Adsorption in SOD-Type Metal–Organic Zeolites. *Chem. Commun.* 2012, *48* (93), 11395–11397. https://doi.org/10.1039/C2CC35544B.

(22) Chen, Z.; Zhang, C.; Liu, X.; Zhang, Z.; Liang, F.; Chen, Z.; Zhang, C.; Liu, X.; Zhang, Z.; Liang, F. Synthesis, Structure, and Properties of a Chiral Zinc(II) Metal-Organic Framework

Featuring Linear Trinuclear Secondary Building Blocks. *Aust. J. Chem.* 2012, *65* (12), 1662–1666. https://doi.org/10.1071/CH12270.

(23) Hou, C.; Liu, Q.; Fan, J.; Zhao, Y.; Wang, P.; Sun, W.-Y. Novel (3,4,6)-Connected Metal-Organic Framework with High Stability and Gas-Uptake Capability. *Inorg Chem* 2012, *51* (15), 8402–8408. https://doi.org/10.1021/ic300950h.

(24) He, J.-H.; Sun, D.-Z.; Xiao, D.-R.; Yan, S.-W.; Chen, H.-Y.; Wang, X.; Yang, J.; Wang, E.-B. Syntheses and Structures of Five 1D Coordination Polymers Based on Quinolone Antibacterial Agents and Aromatic Polycarboxylate Ligands. *Polyhedron* 2012, *42* (1), 24–29. https://doi.org/10.1016/j.poly.2012.04.022.

(25) Guo, M.; Sun, Z.-M. Solvents Control over the Degree of Interpenetration in Metal-Organic Frameworks and Their High Sensitivities for Detecting Nitrobenzene at Ppm Level. *J. Mater. Chem.* 2012, *22* (31), 15939–15946. https://doi.org/10.1039/C2JM32066E.

(26) Shi, D.; Ren, Y.; Jiang, H.; Cai, B.; Lu, J. Synthesis, Structures, and Properties of Two Three-Dimensional Metal-Organic Frameworks, Based on Concurrent Ligand Extension. *Inorg Chem* 2012, *51* (12), 6498–6506. https://doi.org/10.1021/ic202624e.

(27) Kanoo, P.; Ghosh, A. C.; Cyriac, S. T.; Maji, T. K. A Metal–Organic Framework with Highly Polar Pore Surfaces: Selective CO2 Adsorption and Guest-Dependent On/Off Emission Properties. *Chemistry – A European Journal* 2012, *18* (1), 237–244. https://doi.org/10.1002/chem.201101183.

(28) Feng, D.; Gu, Z.-Y.; Li, J.-R.; Jiang, H.-L.; Wei, Z.; Zhou, H.-C. Zirconium-Metalloporphyrin PCN-222: Mesoporous Metal–Organic Frameworks with Ultrahigh Stability as Biomimetic Catalysts. *Angewandte Chemie International Edition* 2012, *51* (41), 10307–10310. https://doi.org/10.1002/anie.201204475.

(29) Wu, P.; Wang, J.; He, C.; Zhang, X.; Wang, Y.; Liu, T.; Duan, C. Luminescent Metal-Organic Frameworks for Selectively Sensing Nitric Oxide in an Aqueous Solution and in Living Cells. *Advanced Functional Materials* 2012, *22* (8), 1698–1703. https://doi.org/10.1002/adfm.201102157.

(30) Schaate, A.; Roy, P.; Preuße, T.; Lohmeier, S. J.; Godt, A.; Behrens, P. Porous Interpenetrated Zirconium–Organic Frameworks (PIZOFs): A Chemically Versatile Family of Metal–Organic Frameworks. *Chemistry – A European Journal* 2011, *17* (34), 9320–9325. https://doi.org/10.1002/chem.201101015.

(31) Liu, D.; Xie, Z.; Ma, L.; Lin, W. Three-Dimensional Metal-Organic Frameworks Based on Tetrahedral and Square-Planar Building Blocks: Hydrogen Sorption and Dye Uptake Studies. *Inorg Chem* 2010, *49* (20), 9107–9109. https://doi.org/10.1021/ic1009169.

(32) Zhuang, W.; Ma, S.; Wang, X.-S.; Yuan, D.; Li, J.-R.; Zhao, D.; Zhou, H.-C. Introduction of Cavities up to 4 Nm into a Hierarchically-Assembled Metal–Organic Framework Using an Angular, Tetratopic Ligand. *Chem. Commun.* 2010, *46* (29), 5223–5225. https://doi.org/10.1039/C0CC00779J.

(33) Barquín, M.; Cocera, N.; González Garmendia, M. J.; Larrínaga, L.; Pinilla, E.; Torres, M. R. Acetato and Formato Copper(II) Paddle-Wheel Complexes with Nitrogen Ligands. *Journal of Coordination Chemistry* 2010, *63* (13), 2247–2260. https://doi.org/10.1080/00958972.2010.502227.

(34) Tian, Y.-Q.; Yao, S.-Y.; Gu, D.; Cui, K.-H.; Guo, D.-W.; Zhang, G.; Chen, Z.-X.; Zhao, D.-Y. Cadmium Imidazolate Frameworks with Polymorphism, High Thermal Stability, and a Large Surface Area. *Chemistry – A European Journal* 2010, *16* (4), 1137–1141. https://doi.org/10.1002/chem.200902729.

(35) Kishan, M. R.; Tian, J.; Thallapally, P. K.; Fernandez, C. A.; Dalgarno, S. J.; Warren, J. E.; McGrail, B. P.; Atwood, J. L. Flexible Metal–Organic Supramolecular Isomers for Gas Separation. *Chem. Commun.* 2010, *46* (4), 538–540. https://doi.org/10.1039/B913910A.

(36) Wang, X.-Z.; Zhu, D.; Xu, Y.; Yang, J.; Shen, X.; Zhou, J.; Fei, N.; Ke, X.; Peng, L. Three Novel Metal-Organic Frameworks with Different Topologies Based on 3,3′-Dimethoxy-4,4′-Biphenyldicarboxylic Acid: Syntheses, Structures, and Properties. 2010. https://doi.org/10.1021/CG9012262.

(37) Hong, S.; Oh, M.; Park, M.; Yoon, J. W.; Chang, J.-S.; Lah, M. S. Large H2 Storage Capacity of a New Polyhedron-Based Metal–Organic Framework with High Thermal and

Hygroscopic Stability. *Chem. Commun.* 2009, No. 36, 5397–5399. https://doi.org/10.1039/B909250A.

(38) Xiang, S.; Zhou, W.; Gallegos, J. M.; Liu, Y.; Chen, B. Exceptionally High Acetylene Uptake in a Microporous Metal-Organic Framework with Open Metal Sites. *J Am Chem Soc* 2009, *131* (34), 12415–12419. https://doi.org/10.1021/ja904782h.

(39) Dai, F.; He, H.; Gao, D.; Ye, F.; Sun, D.; Pang, Z.; Zhang, L.; Dong, G.; Zhang, C. Self-Assembly of 2D Zinc Metal–Organic Frameworks Based on Mixed Organic Ligands. *Inorganica Chimica Acta* 2009, *362* (11), 3987–3992. https://doi.org/10.1016/j.ica.2009.05.038.

(40) Ma, S.; Wang, S.; Collier, C.; Manis, S.; Zhou, H.-C. Ultramicroporous Metal-Organic Framework Based on 9, 10-Anthracenedicarboxylate for Selective Gas Adsorption Inorg. *Inorganic chemistry* 2007, *46*, 8499–8501. https://doi.org/10.1021/ic701507r.

(41) Ma, Y.; Han, Z.; He, Y.; Yang, L. A 3D Chiral Zn(II) Coordination Polymer with Triple Zn–Oba–Zn Helical Chains (Oba = 4,4′-Oxybis(Benzoate)). *Chem. Commun.* 2007, No. 40, 4107–4109. https://doi.org/10.1039/B708479J.

(42) Sun, D.; Ke, Y.; Mattox, T. M.; Parkin, S.; Zhou, H.-C. Stability and Porosity Enhancement through Concurrent Ligand Extension and Secondary Building Unit Stabilization. *Inorg Chem* 2006, *45* (19), 7566–7568. https://doi.org/10.1021/ic0609002.

(43) Sun, D.; Ke, Y.; Collins, D.; Lorigan, G.; Zhou, H.-C. Construction of Robust Open Metal−Organic Frameworks with Chiral Channels and Permanent Porosity. *Inorganic chemistry* 2007, *46*, 2725–2734. https://doi.org/10.1021/ic0624773.

(44) Clausen, H. F.; Poulsen, R. D.; Bond, A. D.; Chevallier, M.-A. S.; Iversen, B. B. Solvothermal Synthesis of New Metal Organic Framework Structures in the Zinc–Terephthalic Acid–Dimethyl Formamide System. *Journal of Solid State Chemistry* 2005, *178* (11), 3342–3351. https://doi.org/10.1016/j.jssc.2005.08.013.

(45) Chun, H.; Dybtsev, D. N.; Kim, H.; Kim, K. Synthesis, X-Ray Crystal Structures, and Gas Sorption Properties of Pillared Square Grid Nets Based on Paddle-Wheel Motifs: Implications for Hydrogen Storage in Porous Materials. *Chemistry – A European Journal* 2005, *11* (12), 3521–3529. https://doi.org/10.1002/chem.200401201.

(46) Adams, H.; Fenton, D. E.; McHugh, P. E. A Heteronuclear [Nickel(II)–Sodium] Infinite Chain Complex Derived from 3-{[(2-Diethylamino-Ethyl)-Methyl-Amino]-Methyl}-2-Hydroxy-5-Methyl-Benzaldehyde. *Inorganic Chemistry Communications* 2004, *7* (7), 880–883. https://doi.org/10.1016/j.inoche.2004.04.025.

(47) Dong, Y.-B.; Ma, J.-P.; Smith, M. D.; Huang, R.-Q.; Tang, B.; Chen, D.; zur Loye, H.-C. New Coordination Polymers Generated from Oxadiazole-Containing Bidentate Ligands and Cu□Cu Dimetal Units. *Solid State Sciences* 2002, *4* (10), 1313–1320. https://doi.org/10.1016/S1293-2558(02)00014-6.

(48) Tao, J.; Yin, X.; Huang, R.; Zheng, L.; Weng Ng, S. Assembly of a Microporous Metal-Organic Framework [Zn(Bpdc)(DMSO)] (Bpdc=4,4′-Biphenyldicarboxylate) Based on Paddle-Wheel Units Affording Guest Inclusion. *Inorganic Chemistry Communications* 2002, *5* (11), 975–977. https://doi.org/10.1016/S1387-7003(02)00623-8.

(49) Papaefstathiou, G. S.; MacGillivray, L. R. An Inverted Metal-Organic Framework with Compartmentalized Cavities Constructed by Using an Organic Bridging Unit Derived from the Solid State. *Angewandte Chemie International Edition* 2002, *41* (12), 2070–2073.

(50) Medishetty R.; Jung D.; Song X.; Kim D.; Lee S. S.; Lah M. S.; Vittal J. J. Solvent-Induced Structural Dynamics in Noninterpenetrating Porous Coordination Polymeric Networks. *Inorg. Chem.* 2013, 52 (6), 2951–2957. https://doi.org/10.1021/ic302334x.

## References

(1) Huang, S.; Cole, J. M. A Database of Battery Materials Auto-Generated Using ChemDataExtractor. *Sci Data* **2020**, *7* (1), 260. https://doi.org/10.1038/s41597-020-00602-2.

(2) Court, C. J.; Cole, J. M. Auto-Generated Materials Database of Curie and Néel Temperatures via Semi-Supervised Relationship Extraction. *Scientific Data* **2018**, *5* (1), 1–12. https://doi.org/10.1038/sdata.2018.111.

(3) Park, S.; Kim, B.; Choi, S.; Boyd, P. G.; Smit, B.; Kim, J. Text Mining Metal–Organic Framework Papers. *J. Chem. Inf. Model.* **2018**, *58* (2), 244–251. https://doi.org/10.1021/acs.jcim.7b00608.

(4) Luo, Y.; Bag, S.; Zaremba, O.; Cierpka, A.; Andreo, J.; Wuttke, S.; Friederich, P.; Tsotsalas, M. MOF Synthesis Prediction Enabled by Automatic Data Mining and Machine Learning. *Angewandte Chemie International Edition* **2022**, *61* (19), e202200242. https://doi.org/10.1002/anie.202200242.
